# A Comparative Real‐World Study Evaluating the Safety of Immune Globulin Infusion (Human) 10% Solution and Other Intravenous Immunoglobulin Therapies for the Treatment of Chronic Inflammatory Demyelinating Polyradiculoneuropathy

**DOI:** 10.1002/pds.70124

**Published:** 2025-03-17

**Authors:** J. Bradley Layton, Colin Anderson‐Smits, Zhongwen Huang, Hakan Ay, William Spalding, Bilal Khokhar, Jie Zhou, Lee Bennett, Mary S. Anthony

**Affiliations:** ^1^ RTI Health Solutions, Research Triangle Park North Carolina USA; ^2^ Takeda Development Center Americas, Inc Cambridge Massachusetts USA

**Keywords:** acute kidney injury, chronic inflammatory demyelinating polyradiculoneuropathy, database study, hemolytic events, immune globulin infusion (human) 10% solution, intravenous immunoglobulin therapies, outcome risk, safety, thrombotic events

## Abstract

**Purpose:**

Immune globulin infusion (human) 10% solution (GAMMAGARD LIQUID; GGL), an intravenous immunoglobulin (IVIG), has recently received U.S. approval for chronic inflammatory demyelinating polyradiculoneuropathy (CIDP). We evaluated thrombotic events, acute kidney injury (AKI), and hemolytic events among patients with CIDP initiating IVIG treatment with GGL, versus a U.S.‐approved IVIG comparator for CIDP, in individual and combined cohorts of immunoglobulin (Ig)‐naive and Ig‐experienced participants.

**Methods:**

This active‐comparator, new‐user, cohort study of patients with CIDP used the Merative MarketScan Research and Optum Clinformatics Data Mart databases (2008–2019). Outcomes were compared between adults receiving GGL and comparator IVIGs within propensity score‐weighted samples using hazard ratios (HRs) and time period‐specific risk ratios (RRs) and risk differences (RDs) with 95% confidence intervals (CI). Database‐specific results were meta‐analyzed and appropriate.

**Results:**

Data from eligible patients in MarketScan (GGL, *n* = 1441; comparators, *n* = 2708) and Optum (GGL, *n* = 644; comparators, *n* = 1293) were analyzed. Across both databases, HRs, 1‐year RRs, and 1‐year RDs for thrombotic events did not suggest consistent differences in risk across treatment groups (e.g., MarketScan combined cohort: RR 1.14 [95% CI, 0.50 to 2.55]; Optum combined cohort: 0.60 [0.25 to 1.54]). Similarly, there was no difference in AKI risk between groups (e.g., MarketScan combined cohort: RR 1.25 [95% CI, 0.65 to 2.54]; Optum combined cohort: 0.65 [0.22 to 1.94]). Hemolytic events were rare.

**Conclusions:**

Thrombotic events, AKI, and hemolytic events were rare among patients with CIDP receiving IVIG. There were no consistently different outcome risks between patients receiving GGL versus other IVIGs with US approval for CIDP.

**Summary**

- We evaluated rates of thrombotic events, acute kidney injury (AKI), and hemolytic events among patients with chronic inflammatory demyelinating polyradiculoneuropathy (CIDP) initiating intravenous immunoglobulin (IVIG) treatment with immune globulin infusion (human) 10% solution (GAMMAGARD LIQUID; GGL) versus those initiating a US‐approved IVIG comparator for CIDP.
- Thrombotic events, AKI, and hemolytic events were rare among patients with CIDP treated with IVIG.
- Comparison of patients treated with GGL and other US‐approved IVIGs for CIDP showed no consistently different risks for the outcomes assessed.

## 1 Introduction

Immune globulin infusion (human) 10% solution (GAMMAGARD LIQUID; GGL) is an intravenous immunoglobulin (IVIG), approved by the US Food and Drug Administration (FDA) in 2005 for patients with primary immunodeficiency disease [1]. In the European Union, GGL is marketed under a different name [2] and has been approved since 2019 to treat chronic inflammatory demyelinating polyradiculoneuropathy (CIDP), a rare inflammatory sensory and motor disorder resulting in substantial functional disability [3]. In January 2024, GGL received US approval for use in CIDP [1]. Other IVIG therapies (such as GAMMAKED or GAMUNEX‐C [both immune globulin injection (human) 10% caprylate/chromatography purified]) and PRIVIGEN (immune globulin intravenous infusion [human], 10% liquid) received US approval for CIDP treatment before GGL. [4, 5, 6] Medical society treatment guidelines for CIDP recommend IVIG treatment but do not differentiate between IVIG therapies [3]. Consequently, GGL has been used off‐label for CIDP in real‐world clinical practice for several years. This analysis was conducted before the US approval of GGL for CIDP and was intended to provide evidence of the comparative safety of GGL and other IVIGs approved for CIDP using real‐world data to support regulatory submission [7].

IVIG therapies carry boxed warnings in the USA for thrombosis, renal dysfunction, and acute renal failure [1, 4, 5, 6]. The purpose of this study was not to refute such warnings, nor to explore the mechanism of action of IVIG in CIDP, which is complex and the subject of ongoing investigation; broadly, however, the effect is an immunomodulatory mitigation of peripheral nerve demyelination [8]. This is achieved with high doses (e.g., 1.0 g/kg bodyweight) of immunoglobulins relative to the dose levels typically used in immunoglobulin replacement therapies for immunodeficiencies (e.g., 0.3–0.6 g/kg) [1, 4, 5, 6]. For this reason, patients with CIDP receiving IVIG may have different safety outcomes than patients receiving IVIG for other diseases.

The safety of IVIG therapies as a class has been evaluated in US administrative claims databases, but study populations were not restricted to participants with CIDP [9, 10, 11, 12]. A number of previous studies examined the comparative risks across IVIG therapies using GGL as the reference group (because GGL was the most frequently used IVIG) and suggested that the risk of some safety outcomes may vary by therapy [10, 11, 12, 13]. Given the reported off‐label clinical use of GGL for CIDP treatment at the time of the study, more data were needed on the comparative safety of GGL and other IVIGs when used in patients with CIDP.

This observational study evaluated rates of thrombotic events, acute kidney injury (AKI), and hemolytic events among patients with CIDP initiating IVIG treatment with GGL versus those initiating a comparator IVIG, in cohorts of immunoglobulin (Ig)‐naive and Ig‐experienced participants.

## 2 Methods

### 2.1 Setting

This was an active‐comparator, new‐user, cohort study of participants with CIDP initiating treatment with GGL or one of the following comparator IVIGs, indicated for CIDP treatment: immune globulin injection (human) 10%, caprylate/chromatography purified (GAMMAKED, GAMUNEX‐C) or immune globulin intravenous (human) 10% liquid (PRIVIGEN). This study was conducted using two US‐based administrative insurance claims databases: the Merative MarketScan Research Databases (including commercial, Medicare supplementary, and Medicaid insurance data) and the Optum Clinformatics Data Mart (Optum; including commercial and Medicare supplementary insurance data). Given the rarity of CIDP and the outcomes of interest, two databases were used to maximize the number of IVIG‐treated patients identified. These sources consist of longitudinal enrollment information and billing claims for enrollees, spouses, and dependents. The study protocol was registered prior to study initiation at the US Clinical Trials registry (NCT05363358) and the European Union electronic Register of Post‐Authorisation Studies Network of Centres for Pharmacoepidemiology and Pharmacovigilance (EUPAS46101).

IVIG initiators were identified between January 1, 2008 (the year of initial FDA approval for IVIG use in CIDP [GAMUNEX‐C] [14]) and December 31, 2019 (prior to COVID‐19 pandemic‐related healthcare disruptions [15]). Additionally, data back to January 1, 2001 in MarketScan and January 1, 2000 in Optum, if available, were used for baseline variable and eligibility assessments.

### 2.2 Participants

Participants aged at least 18 years were identified at the initiation of one of the study IVIGs (the index date). Two cohorts were identified: (1) participants newly initiating an IVIG with no record of previous use of any Ig therapy (Ig‐naive cohort) and (2) participants newly initiating either GGL or a comparator IVIG but with previous use of any other Ig therapy (Ig‐experienced cohort); only the first eligible IVIG initiation in an Ig‐experienced participant was included. Participants in the Ig‐naive cohort could also be eligible for inclusion in the Ig‐experienced cohort if they switched IVIG therapy.

To be eligible for either cohort, identified participants were required to have a minimum of 183 days of continuous enrollment in a study database with medical and pharmacy coverage before IVIG initiation (gaps in continuous enrollment of 31 days or less were permitted) and at least two recorded CIDP diagnoses separated by at least 14 days on or before the date of a qualifying IVIG initiation. Participants were excluded if they had claims for two different Ig therapies on the index date, had a recorded diagnosis of other conditions typically treated with IVIG, or were missing a designation for sex in the enrollment information (Data S1, Figure S1).

For each outcome‐specific analysis, follow‐up began on the index date and ended at the occurrence of the outcome or censoring at the first occurrence of any of the following events: end of the study period (December 31, 2019), disenrollment from continuous eligible medical and/or pharmacy coverage, discontinuation of the index IVIG, or switching to or adding a different Ig therapy or a brand‐unspecified Ig treatment.

### 2.3 Exposure Assessment

IVIG use was identified in the data using procedure coding for IVIG administration or pharmacy dispensing records for IVIGs [16]. Participants were considered to have discontinued the IVIG if they failed to receive another dose within 9 weeks of the previous dose (typical dosing is every 3 weeks; however, 9 weeks were allowed to account for skipped or delayed doses).

### 2.4 Outcome Assessment

Outcomes were identified during follow‐up using International Classification of Diseases, Ninth Edition, Clinical Modification and Tenth Edition diagnosis codes from submitted claims from inpatient, emergency department, or outpatient providers. Where available, this study implemented validated claims‐based algorithms for the outcomes of interest used in other studies of IVIG users. Primary outcomes included thrombotic events (composite of acute ischemic stroke [17], acute myocardial infarction [18], and acute venous thromboembolism [VTE] [19]), AKI [12], and hemolytic events (Table S1). The occurrence of primary outcome events was expected within days to weeks of IVIG administration [20]. Only the first occurrence of each outcome during follow‐up was considered, and analyses of individual outcomes were performed separately in outcome‐specific analysis sets created by applying outcome‐specific exclusion criteria. Secondary outcomes, including anaphylaxis, transfusion‐related acute lung injury, and transfusion‐associated circulatory overload, were also assessed, though sample sizes (number of events) were too small for meaningful analyses.

### 2.5 Statistical Methods

Separate analyses were performed for each cohort and database for each outcome. Outcome‐specific propensity scores were estimated in each cohort using logistic regression with prespecified, outcome‐specific baseline covariates (Table S2). Propensity scores were transformed into matching weights [21, 22]. The relative balance of patient characteristics between treatment groups before and after weighting was described with absolute standardized differences [23, 24].

Incidence rates (IRs) and exact 95% confidence intervals (CIs) [25] were estimated for each outcome across follow‐up. Cumulative incidence curves were generated by treatment group as one minus the Kaplan–Meier survival estimator. Risk ratios (RRs) and risk differences (RDs) were estimated from the cumulative incidence at day 365 and at shorter intervals (days 0, 3, 14, 30, and 90) by dividing or subtracting, respectively, treatment group‐specific risk estimates at each time interval; 95% CIs were estimated using nonparametric bootstrapping [26, 27].

Hazard ratios (HRs) and 95% CIs were estimated using weighted Cox proportional hazards models with robust sandwich‐style variance estimators. The proportional hazards assumption was assessed with a visual evaluation of hazard functions, log–log survival curves, and statistical testing of goodness‐of‐fit using Schoenfeld residuals [23]. If appropriate, database‐specific estimates were meta‐analyzed using fixed‐effects meta‐analytic methods (Data S1). Weighted HRs and 95% CIs were also estimated in subgroups of sex, age group, and pre‐existing renal disease.

### 2.6 Sensitivity and Quantitative Bias Analyses

As a sensitivity analysis, the assumed duration of IVIG exposure was extended from 9 to 12 weeks to observe late‐occurring adverse events (AEs). An additional sensitivity analysis modified the inclusion criteria so that only a single diagnosis of CIDP was required. A quantitative bias analysis considered a range of differential outcome misclassification scenarios to estimate the extent of misclassification necessary to substantively alter the study conclusions (Data S1 and Figures S2–S6) [28, 29].

## 3 Results

### 3.1 Participant Characteristics

**FIGURE 1:**
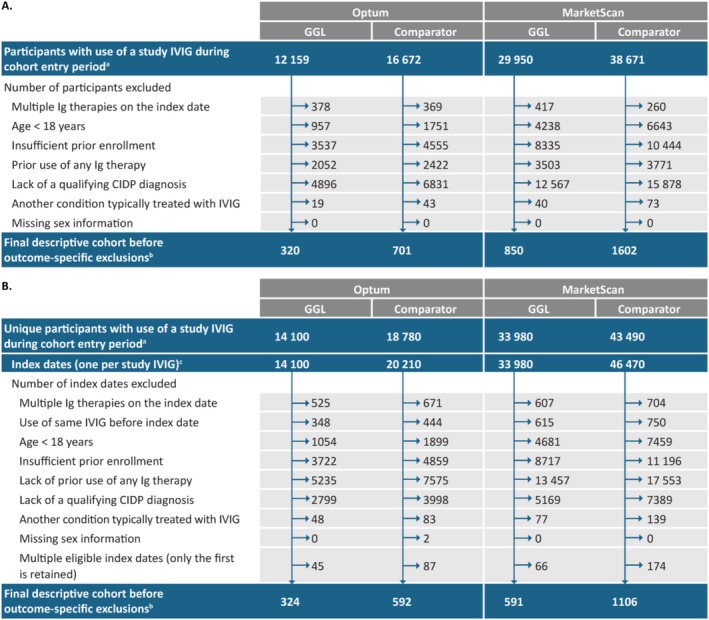
Participant exclusion criteria for the Ig‐naive (a) and Ig‐experienced (b) cohorts. ^a^The cohort entry period was January 1, 2008, to December 31, 2019. ^b^To generate outcome‐specific analysis sets, outcome‐specific exclusion criteria were separately applied to the descriptive cohorts. ^c^For the comparator cohort, participants initiating more than one IVIG had additional index dates. CIDP, chronic inflammatory demyelinating polyradiculoneuropathy; GGL, immune globulin infusion (human) 10% solution (GAMMAGARD LIQUID); Ig, immunoglobulin; IVIG, intravenous immunoglobulin.

There were 1441 GGL and 2708 comparator IVIG users identified in the MarketScan combined cohort (either Ig‐naive or Ig‐experienced), and 644 GGL and 1293 comparator users in Optum (Figure 1). Across all cohorts and databases, participants were predominantly male (≥ 54%) and the age range across both databases spanned 8 years to more than 90 years. Median (first quartile, third quartile) duration of use of GGL and comparator IVIGs since index was 108 (64, 247) days and 111 (64, 258) days, respectively, in MarketScan, and 111 (64, 253) days and 115 (64, 255) days, respectively, in Optum. Participants in each cohort generally had a high burden of serious comorbidities, comedications, or previous CIDP treatments, indicators for CIDP severity or compromised functional status, and markers of CIDP diagnostic workup (Table 1). Additionally, comorbidities such as hypertension, dyslipidemia, and diabetes were higher in Optum than in MarketScan, consistent with the older age distribution of patients in Optum (Table 1).

**TABLE 1:** Select characteristics of IVIG initiators with CIDP in the combined cohort, by database and treatment group.

Characteristic	Optum		MarketScan	
	GGL *N* = 644	Comparator *N* = 1293	GGL *N* = 1441	Comparator *N* = 2708
Age, years				
Mean (SD)	61.2 (14.0)	61.5 (15.1)	56.9 (13.8)	55.8 (14.2)
Median [Q1, Q3]	62 [52, 72]	63 [52, 73]	58 [49, 65]	57 [48, 64]
Sex, *n* (%)				
Female	257 (39.9)	504 (39.0)	619 (43.0)	1171 (43.2)
Male	387 (60.1)	789 (61.0)	822 (57.0)	1537 (56.8)
Geographic region, *n* (%)				
Midwest	142 (22.0)	241 (18.6)	327 (22.7)	359 (13.3)
Northeast	90 (14.0)	183 (14.2)	303 (21.0)	556 (20.5)
South	291 (45.2)	626 (48.4)	500 (34.7)	1139 (42.1)
West	121 (18.8)	243 (18.8)	185 (12.8)	328 (12.1)
Unknown	0 (0.0)	0 (0.0)	126 (8.7)	326 (12.0)
Calendar year of index date, *n* (%)				
2008	59 (9.2)	67 (5.2)	141 (9.8)	114 (4.2)
2009	47 (7.3)	53 (4.1)	94 (6.5)	142 (5.2)
2010	31 (4.8)	64 (4.9)	168 (11.7)	213 (7.9)
2011	41 (6.4)	68 (5.3)	165 (11.5)	268 (9.9)
2012	44 (6.8)	116 (9.0)	163 (11.3)	377 (13.9)
2013	37 (5.7)	74 (5.7)	129 (9.0)	249 (9.2)
2014	36 (5.6)	88 (6.8)	147 (10.2)	289 (10.7)
2015	37 (5.7)	80 (6.2)	97 (6.7)	201 (7.4)
2016	58 (9.0)	126 (9.7)	83 (5.8)	236 (8.7)
2017	81 (12.6)	167 (12.9)	90 (6.2)	220 (8.1)
2018	91 (14.1)	194 (15.0)	83 (5.8)	222 (8.2)
2019	82 (12.7)	196 (15.2)	81 (5.6)	177 (6.5)
Hypertension, *n* (%)	456 (70.8)	940 (72.7)	901 (62.5)	1673 (61.8)
Lipid abnormality, *n* (%)	465 (72.2)	915 (70.8)	836 (58.0)	1554 (57.4)
Diabetes mellitus, *n* (%)	281 (43.6)	494 (38.2)	488 (33.9)	919 (33.9)
High‐dose, systemic corticosteroid use, *n* (%)	390 (60.6)	790 (61.1)	849 (58.9)	1627 (60.1)

*Note:* A participant may appear in the combined cohort more than once if they were included in both the Ig‐naive and Ig‐experienced cohorts. Therefore, the unit of analysis for the combined cohort is the combination of participant and index date. The *N* displayed is the count of unique participant–index date combinations; the participants displayed in the table were summarized for each index date.

Abbreviations: CIDP, chronic inflammatory demyelinating polyradiculoneuropathy; GGL, immune globulin infusion (human) 10% solution (GAMMAGARD LIQUID); Ig, immunoglobulin; IVIG, intravenous immunoglobulin; Q1, Q3, first and third quartiles; SD, standard deviation.

**FIGURE 2:**
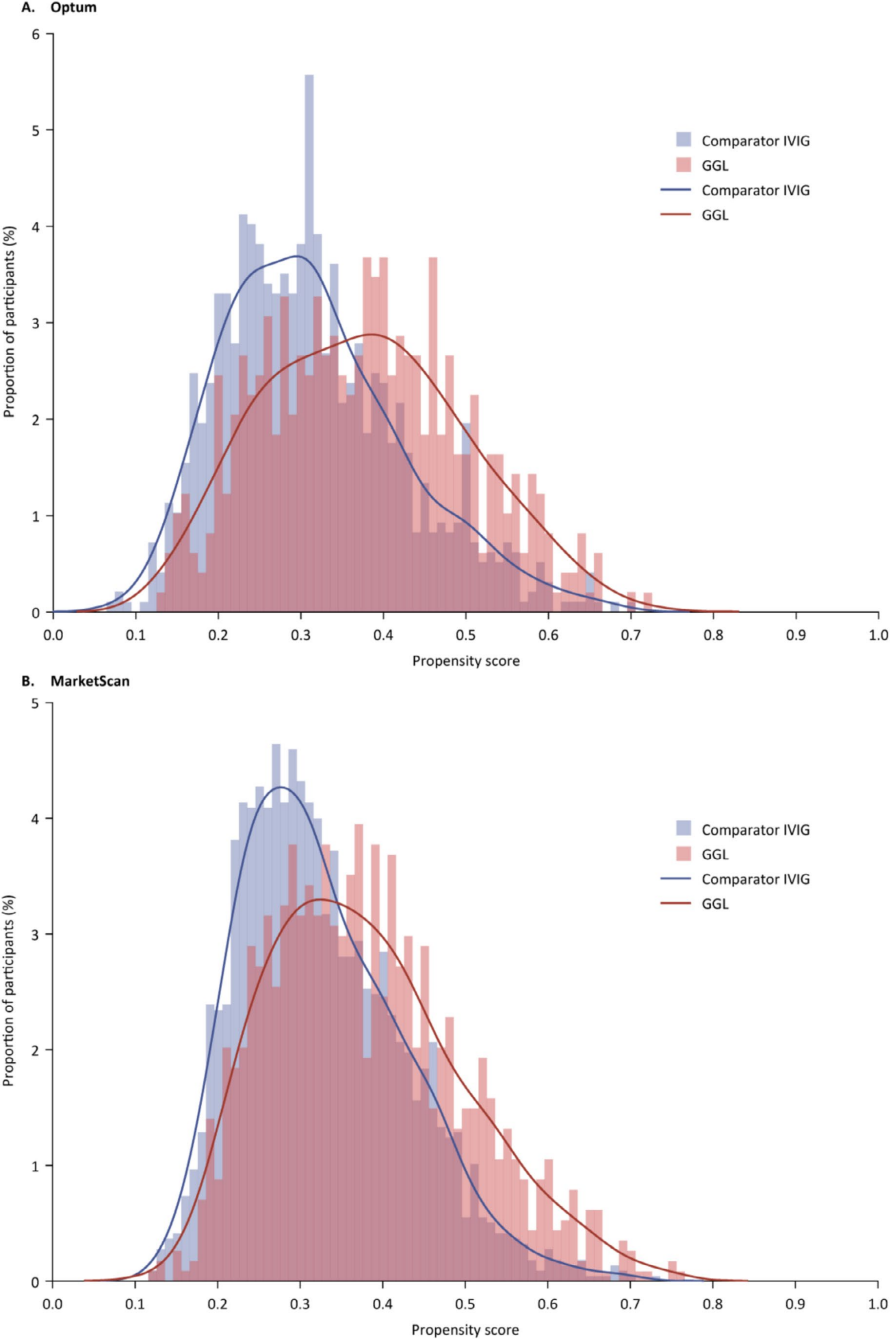
Distribution of propensity scores of GGL and comparator IVIG initiators with CIDP by database; combined cohorts, thrombotic events analysis set. CIDP, chronic inflammatory demyelinating polyradiculoneuropathy; GGL, immune globulin infusion (human) 10% solution (GAMMAGARD LIQUID); IVIG, intravenous immunoglobulin.

While differences in calendar year and US geographic region between the GGL and comparator groups were noted across all cohorts (Table 1), few meaningful differences in clinical characteristics were found between treatment groups. The propensity score overlap in each outcome‐specific analysis set indicated good overlap and exchangeability between treatment groups (an example for thrombotic events in the combined cohort is shown in Figure 2). An example of the unweighted and weighted absolute standardized differences of the covariates for the thrombotic events composite outcome analysis set for the combined Ig‐naive and ‐experienced cohort in the Optum database is shown in Figure S7.

### 3.2 Primary Outcomes

**FIGURE 3:**
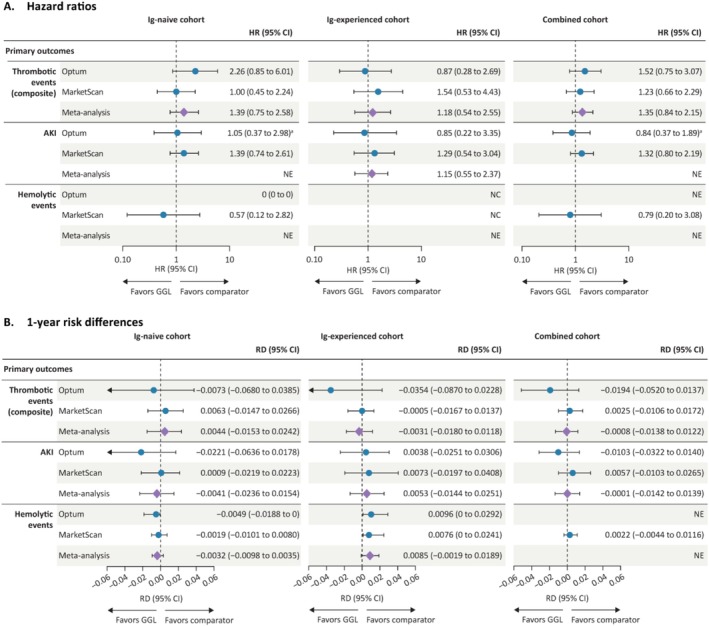
Propensity score‐weighted (A) hazard ratios and (B) risk differences for thrombotic events, AKI, and hemolytic events with GGL versus comparator IVIGs across cohorts and databases. ^a^Assumption of proportional hazards was violated; *p* < 0.05 for the proportional hazards assumption test of goodness‐of‐fit using Schoenfeld residuals on the crude model. AKI, acute kidney injury; CI, confidence interval; GGL, immune globulin infusion (human) 10% solution (GAMMAGARD LIQUID); HR, hazard ratio; Ig, immunoglobulin; IVIG, intravenous immunoglobulin; NC, not calculable; NE, not estimated (analysis not attempted because it did not meet protocol‐specified criteria); RD, risk difference.

The key results of the propensity score–weighted HRs and 1‐year RDs for primary outcomes by database and cohort are shown in Figure 3.

#### 3.2.1 Thrombotic Events

**FIGURE 4:**
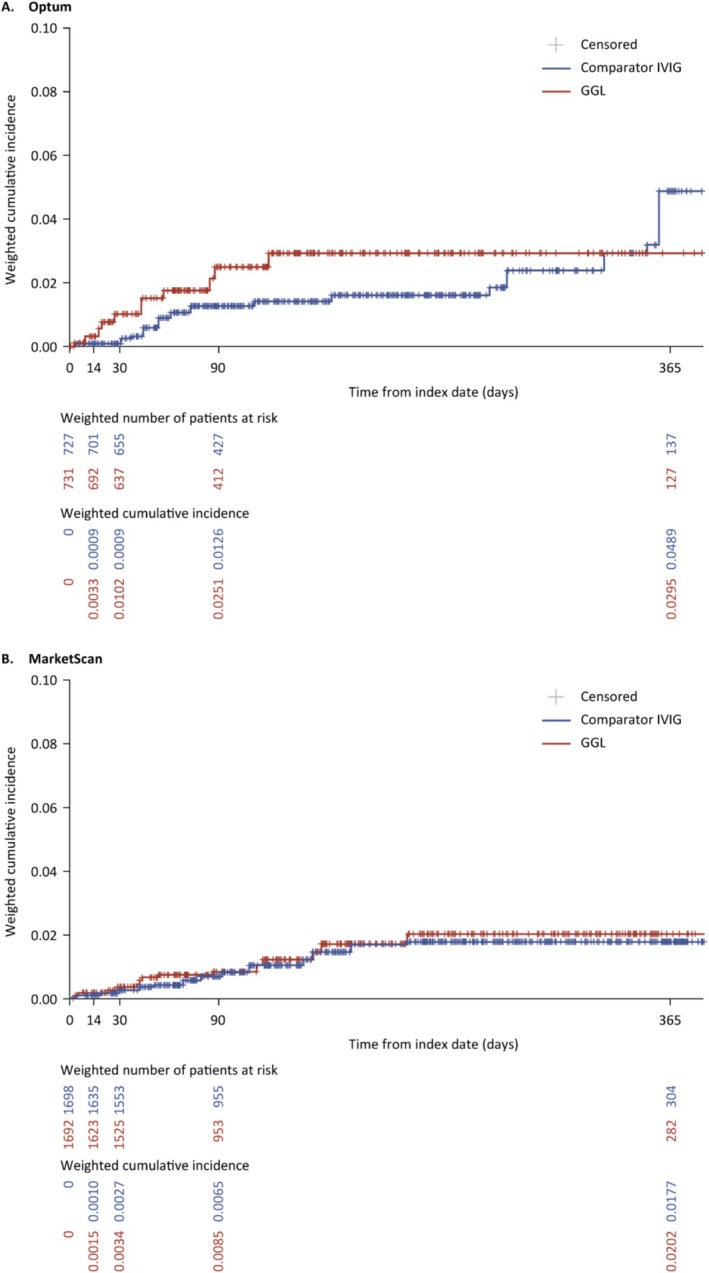
Weighted cumulative incidence of thrombotic events in IVIG initiators with CIDP by treatment group and database, combined cohort. CIDP, chronic inflammatory demyelinating polyradiculoneuropathy; GGL, immune globulin infusion (human) 10% solution (GAMMAGARD LIQUID); IVIG, intravenous immunoglobulin.

In Optum, the majority of the identified thrombotic events in GGL users occurred within the first 90 days of treatment (i.e., the weighted risk was approximately 2.5% [10 events] on day 90 and 3.0% on day 365 (11 events; Figure 4); however, for comparator IVIG users, most of the events occurred later in the period (e.g., the weighted risk was approximately 1.3% [8 events] on day 90 and 4.9% [16 events] on day 365). While the early separation of the cumulative incidence curves (Figure 4A) resulted in an HR point estimate of 1.52 (95% CI, 0.75 to 3.07; Table 2), the later‐occurring events in the comparator group resulted in a day 365 RR below 1 (0.60; 95% CI, 0.25 to 1.54) and an RD below 0 (−0.0194; 95% CI, −0.0520 to 0.0137), although both were imprecise.

**TABLE 2:** Association of initiating GGL with thrombotic events in participants with CIDP, compared with those initiating other IVIGs by data set, combined cohort.

Treatment group	*N*	Person‐years	Events	Crude IR (95% CI)^a^	Crude HR (95% CI)	Weighted HR (95% CI)^b^	Weighted 1‐year RR (95% CI)^b^	Weighted 1‐year RD (95% CI)^b^
Optum								
GGL	489	314.1	15	47.8 (26.7 to 78.8)	1.68 (0.85 to 3.34)	1.52 (0.75 to 3.07)	0.60 (0.25 to 1.54)	−0.0194 (−0.0520 to 0.0137)
Comparator	969	623.6	18	28.9 (17.1 to 45.6)	—	—	—	—
MarketScan								
GGL	1165	740.0	17	23.0 (13.4 to 36.8)	1.07 (0.59 to 1.95)	1.23 (0.66 to 2.29)	1.14 (0.50 to 2.55)	0.0025 (−0.0106 to 0.0172)
Comparator	2225	1380.3	30	21.7 (14.7 to 31.0)	—	—	—	—

*Note:* — indicates the reference group.

Abbreviations: CI, confidence interval; CIDP, chronic inflammatory demyelinating polyradiculoneuropathy; GGL, immune globulin infusion (human) 10% solution (GAMMAGARD LIQUID); HR, hazard ratio; IVIG, intravenous immunoglobulin; IR, incidence rate; RD, risk difference; RR, risk ratio.

^a^ IR estimates scaled as events per 1000 person‐years.

^b^ Propensity score weighted.

In MarketScan, the occurrence of thrombotic events over time was very similar across both treatment groups (Figure 4B), resulting in a HR of 1.23 (95% CI, 0.66 to 2.29), a 1‐year RR of 1.14 (95% CI, 0.50 to 2.55) and a 1‐year RD of 0.0025 (95% CI, −0.0106 to 0.0172; Table 2). Analyses of the individual components of the thrombotic events composite are shown in Data S1. In supplementary analysis, no included participants had a VTE occurrence on the index date. All VTE cases were observed from 3 days post‐index within both datasets.

#### 3.2.2 Acute Kidney Injury

In Optum, the overall weighted HR for AKI in the combined cohort failed the assumption of proportional hazards. The 1‐year RR and RD point estimates were below the null; the 1‐year RR was 0.65 (95% CI, 0.22 to 1.94) and the 1‐year RD was −0.0103 (95% CI, −0.0322 to 0.0140; Table 3). In MarketScan, all point estimates were just above the null; the HR was 1.32 (95% CI, 0.80 to 2.19), the 1‐year RR was 1.25 (95% CI, 0.65 to 2.54), and the 1‐year RD was 0.0057 (95% CI, −0.0103 to 0.0265). The weighted cumulative incidence curves for AKI for the combined cohort are shown in (Figure S8). Owing to the failed assumption of proportionality, the combined cohort HRs were not meta‐analyzed across databases.

**TABLE 3:** Association of initiating GGL with acute kidney injury in participants with CIDP, compared with those initiating other IVIGs by data set, combined cohort.

Treatment group	*N*	Person‐years	Events	Crude IR (95% CI)^a^	Crude HR (95% CI)	Weighted HR (95% CI)^b^	Weighted 1‐year RR (95% CI)^b^	Weighted 1‐year RD (95% CI)^b^
Optum								
GGL	563	362.3	9	24.8 (11.4 to 47.2)	0.86 (0.39 to 1.88)^c^	0.84 (0.37 to 1.89)^c^	0.65 (0.22 to 1.94)	−0.0103 (−0.0322 to 0.0140)
Comparator	1109	702.9	21	29.9 (18.5 to 45.7)	—	—	—	—
MarketScan								
GGL	1302	848.2	28	33.0 (21.9 to 47.7)	1.29 (0.80 to 2.09)	1.32 (0.80 to 2.19)	1.25 (0.65 to 2.54)	0.0057 (−0.0103 to 0.0265)
Comparator	2426	1480.4	40	27.0 (19.3 to 36.8)	—	—	—	—

*Note:* — indicates the reference group.

Abbreviations: CI, confidence interval; CIDP, chronic inflammatory demyelinating polyradiculoneuropathy; GGL, immune globulin infusion (human) 10% solution (GAMMAGARD LIQUID); HR, hazard ratio; Ig, immunoglobulin; IVIG, intravenous immunoglobulin; IR, incidence rate; RD, risk difference; RR, risk ratio.

^a^ IRs scaled as events per 1000 person‐years.

^b^ Propensity score weighted.

^c^ Assumption of proportional hazards was violated; *p* < 0.05 for the proportional hazards assumption test of goodness‐of‐fit using Schoenfeld residuals on the crude model.

#### 3.2.3 Hemolytic Events

Overall, hemolytic events were rare (Table 4). In Optum, HRs and RRs for the separate Ig‐naive and Ig‐experienced cohorts could not be estimated, and the combined cohort was not analyzed owing to evidence of heterogeneity. In the MarketScan combined cohort, the HR for hemolytic events was 0.79 (95% CI, 0.20 to 3.08), the 1‐year RR was 1.57 (95% CI could not be estimated), and the 1‐year RD was 0.0022 (95% CI, −0.0044 to 0.0116). Weighted cumulative incidence curves for hemolytic events for the Ig‐naive and Ig‐experienced cohorts are shown in Figure S9.

**TABLE 4:** Association of initiating GGL with hemolytic events in participants with CIDP, compared with those initiating other IVIGs by database and cohort.

Cohort	Treatment group	*N*	Person‐years	Events	Crude IR (95% CI)^a^	Crude HR (95% CI)	Weighted HR (95% CI)^b^	Weighted 1‐year RR (95% CI)^b^	Weighted 1‐year RD (95% CI)^b^
Optum									
Ig‐naive	GGL	313	178.9	0	0 (0 to 20.6)	0 (0 to NC)	0 (0 to 0)	NC (NC to NC)	−0.0049 (−0.0188 to 0)
	Comparator	677	422.5	3	7.1 (1.5 to 20.8)	—	—	—	—
Ig‐experienced	GGL	321	228.9	2	8.7 (1.1 to 31.6)	NC	NC	NC	0.0096 (0 to 0.0292)
	Comparator	579	368.1	0	0 (0 to 10.0)	—	—	—	—
MarketScan									
Combined	GGL	1413	912.5	3	3.3 (0.7 to 9.6)	0.71 (0.19 to 2.69)	0.79 (0.20 to 3.08)	1.57 (NC to NC)	0.0022 (−0.0044 to 0.0116)
	Comparator	2658	1619.2	8	4.9 (2.1 to 9.7)	—	—	—	—

*Note:* — indicates the reference group.

Abbreviations: CI, confidence interval; CIDP, chronic inflammatory demyelinating polyradiculoneuropathy; GGL, immune globulin infusion (human) 10% solution (GAMMAGARD LIQUID); HR, hazard ratio; Ig, immunoglobulin; IVIG, intravenous immunoglobulin; IR, incidence rate; NC, not calculable (analysis attempted, per protocol, but analysis failed); RD, risk difference; RR, risk ratio.

^a^ IRs scaled as events per 1000 person‐years.

^b^ Propensity score weighted.

### 3.3 Other Analyses

#### 3.3.1 Subgroup Analyses

**FIGURE 5:**
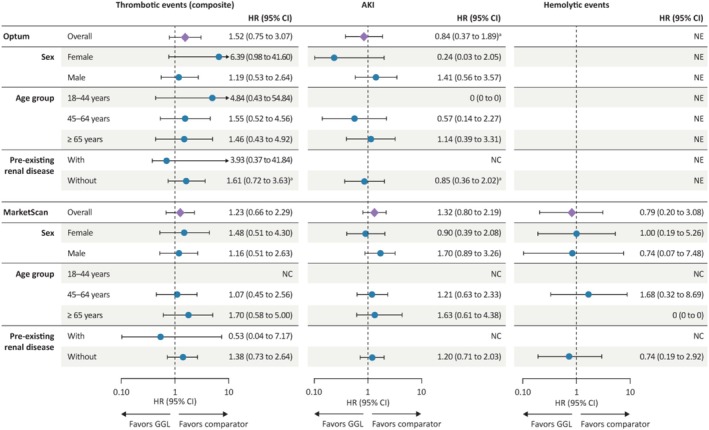
Propensity score‐weighted hazard ratios for thrombotic events, AKI, and hemolytic events with GGL versus comparator IVIGs by data set and subgroup for combined cohorts. ^a^Assumption of proportional hazards was violated; *p* < 0.05 for the proportional hazards assumption test of goodness‐of‐fit using Schoenfeld residuals on the crude model. AKI, acute kidney injury; CI, confidence interval; CIDP, chronic inflammatory demyelinating polyradiculoneuropathy; GGL, immune globulin infusion (human) 10% solution (GAMMAGARD LIQUID); IVIG, intravenous immunoglobulin; NE, not estimated (analysis not attempted because it did not meet protocol‐specified criteria.

Subgroup analyses resulted in smaller sample sizes and less precise estimates, but some small numeric differences were noted across some subgroups and databases (Figure 5; Data S1).

#### 3.3.2 Sensitivity Analyses

**FIGURE 6:**
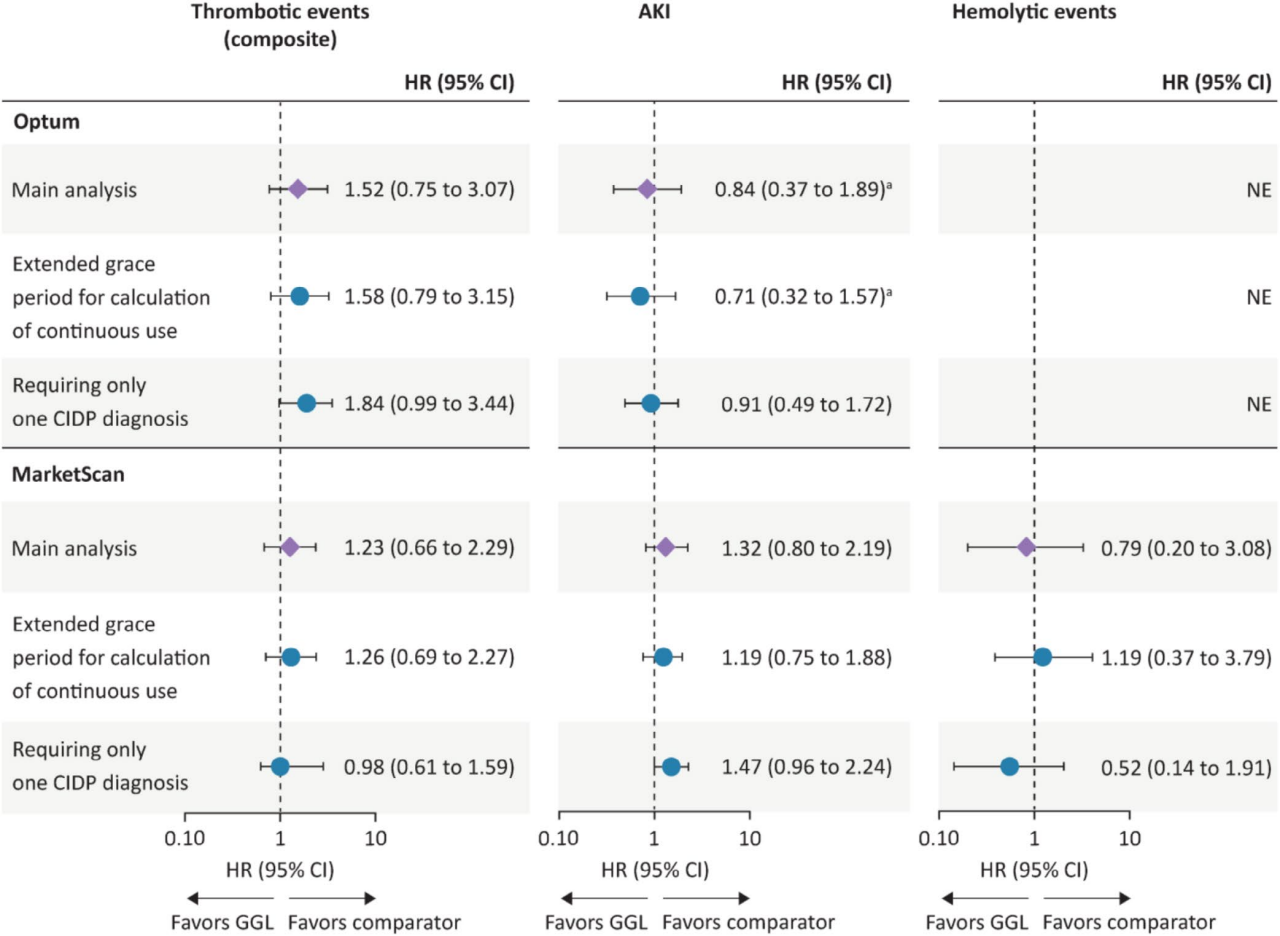
Propensity score‐weighted hazard ratios for thrombotic events, AKI, and hemolytic events with GGL versus comparator IVIGs for the main and sensitivity analyses for combined cohorts by data set. ^a^Assumption of proportional hazards was violated; *p* < 0.05 for the proportional hazards assumption test of goodness‐of‐fit using Schoenfeld residuals on the crude model. AKI, acute kidney injury; CI, confidence interval; CIDP, chronic inflammatory demyelinating polyradiculoneuropathy; GGL, immune globulin infusion (human) 10% solution (GAMMAGARD LIQUID); IVIG, intravenous immunoglobulin; NE, not estimated (analysis not attempted because it did not meet protocol‐specified criteria.

Extending the assumed IVIG duration from 9 to 12 weeks resulted in no notable differences versus the main analysis (Figure 6). In the sensitivity analysis requiring only one CIDP diagnosis, despite the increased sample size, most resulting HRs were imprecise and compatible with HRs from the main analysis. For thrombotic events in Optum, weighted HRs from the sensitivity analysis were higher than those from the main analysis, but in MarketScan, HRs were lower than those from the main analysis.

#### 3.3.3 Quantitative Bias Analysis

The quantitative bias analysis indicated that few conclusions from the main analyses would be changed at reasonable levels of differential outcome misclassification. However, for outcomes with RRs from the main analysis close to the null or slightly above the null (e.g., thrombotic events in MarketScan [RR = 1.14] or AKI in MarketScan [RR = 1.25]), differential misclassification of 20% could potentially result in a true RR of greater than 1.5 (Data S1 and Figures S2–S6).

## 4 Discussion

More than 30% of US patients with CIDP identified for the study initiated IVIG treatment with GGL. In both databases and cohorts, the characteristics of GGL and comparator IVIG initiators were closely comparable, suggesting that GGL and other IVIGs were used very similarly.

Identified events of the study outcomes (thrombotic events, AKI, and hemolytic events) were rare for almost all outcomes in all cohorts. However, when comparing the risk of thrombotic events in GGL users with users of other IVIG therapies, the results were slightly inconsistent across databases and over time. In MarketScan, estimates for the two groups were similar throughout follow‐up; in Optum, we observed more thrombotic events early after treatment initiation among the GGL group than among the comparator group (contributing to an increased HR point estimate, though with somewhat wide CIs) but a lower overall risk after 1 year (contributing to RR and RD estimates below the null, though CIs were also wide). This study evaluated a treatment strategy of IVIG initiation and continuing treatment with repeat IVIG administrations every few weeks, rather than events occurring after a single administration. Therefore, events occurring during follow‐up were identified relative to treatment initiation, though they may have occurred after multiple administrations during follow‐up. These observed differences at different time points after treatment initiation were often driven by a small number of events, requiring caution in interpretation. The reasons for these differences between databases are unclear, but may result from the low number of events overall; differences in patient characteristics by IVIG therapy were not expected (the insurance provider supplying the Optum data does not differentiate between IVIGs in its policies [30]), and differences in measured patient characteristics were accounted for after propensity score weighting. Although an early increased risk of thrombotic events associated with GGL use cannot be excluded in this study (as it may be indicative of a clinically relevant risk which is difficult to observe owing to a limited number of events), the magnitude of the absolute difference between groups would likely be small.

Thrombosis is a recognized potential complication of IVIG use [10, 11]. Our study was restricted to IVIG initiators with CIDP, and while sample sizes were small, it did not indicate any consistent differences in thrombosis risk between GGL and other IVIGs indicated for CIDP. While the results of some analyses using certain databases are imprecise, there might be slight differences in risk that are time dependent. Potentially higher early HRs still correlated to very low differences in overall absolute risk identified with the RDs.

Comparisons of thrombotic events in our study with those previously published are difficult because the present study examined repeated exposure to IVIGs during continuous periods of treatment (median > 100 days), whereas follow‐up periods and dosing regimens used in other studies vary widely [10, 12, 13, 31]. A previous FDA study of IVIG users with any indication evaluated arterial thromboembolisms (acute ischemic stroke and acute myocardial infarction) using a self‐controlled design with defined risk‐windows, but only considered time within 2 days of IVIG administration (for a single dose) or 2 days after the final dose (if IVIG was administered over 3 consecutive days) [31], in contrast to the repeated exposure examined here. Also, a UK study of IVIG use among patients with inflammatory neuropathies reported VTE risks based on only one case [32].

Previous FDA studies of same‐day hemolytic reactions and AKI associated with Ig (intravenous or subcutaneous; not restricted to patients with CIDP) suggested that GGL was not associated with differences in hemolysis and AKI risks as for other IVIGs [12, 13]. The IRs of hemolysis or AKI observed in the present study cannot be compared with those observed in the FDA studies because those studies were restricted to events occurring on the same day as Ig administration and reported risks (events per person) rather than IRs over time. However, the overall conclusions of our study align with those observed in the FDA studies.

The balance of measured clinical characteristics between treatment groups, even before any statistical adjustment, suggests limited measured confounding. Some differences by calendar year and US geographic region were noted, likely resulting from IVIG availability fluctuations over time. Although these and other demographic factors were adjusted for in propensity score models, unmeasured confounding by characteristics not recorded in claims data could not be excluded. For example, over time, physicians may have become aware of the association between CIDP treatment and thrombotic events, potentially influencing their treatment decisions. An additional limitation is that while the individual components of the thrombotic events composite have been validated, the composite measure itself has not been validated.

The new‐user, active‐comparator design can be expected to reduce the potential for bias in nonrandomized studies by aligning the treatment and comparator groups at equivalent points in their disease and treatment histories and on key factors, including healthcare‐seeking behavior and access [33]. Additionally, selection bias was addressed by ensuring that eligibility criteria were applied equally to all individuals in both the GGL and comparator groups in both cohorts [34].

## 5 Conclusion

Thrombotic events, AKI, and hemolytic events were rare among patients with CIDP treated with IVIG. There was no consistent difference in risk of the study outcomes between those treated with GGL and those treated with other IVIGs approved for use in patients with CIDP in the USA. In Optum, we observed more thrombotic events early after treatment initiation among the GGL group than the comparator group. Owing to the imprecision of some estimates, increased risks of small absolute magnitude cannot be ruled out by this study; however, the absolute difference between the groups was very small and varied over time.

### 5.1 Plain Language Summary

Intravenous immunoglobulin (IVIG) is an antibody therapy recommended to treat a rare autoimmune disease called chronic inflammatory demyelinating polyradiculoneuropathy (CIDP). Immune globulin infusion (human) 10% solution (GAMMAGARD LIQUID; GGL) is a type of IVIG treatment recently approved for CIDP in the USA. GGL was not approved for CIDP treatment at the time of this study but had been used by patients with the disease before approval. Our study aimed to assess the safety of GGL compared with other US‐approved IVIG treatments in patients with CIDP. Information on adverse events during treatment was collected from health insurance databases between 2008 and 2019 and consisted of records of patients who were diagnosed with CIDP. We compared the rates of three outcomes: diseases caused by a blood clot (thrombotic events), reduced kidney function (acute kidney injury), and abnormal breakdown of red blood cells (hemolytic events). These outcomes were all rare among the patients included in this study. Statistical analyses to compare the risk of these events happening showed no consistent difference between treatments. In conclusion, a low number of the adverse events described above occurred in patients with CIDP, and the related risks were generally similar between patients receiving different IVIG therapies and GGL.

## Author Contributions

*Conception of the work*: **J.B.L., C.A.‐S., W.S**., and **B.K** . *Design of the work*: **J.B.L., C.A.‐S., W.S., B.K**., and **M.S.A.** *Data acquisition*: **Z.H.** and **W.S.** *Statistical/data analysis*: **J.B.L**., **C.A.‐S., Z.H., W.S., B.K., J.Z**., and **M.S.A.** *Interpretation of data*: **J.B.L., C.A.‐S., Z.H., W.S., B.K., J.Z., L.B**., and **M.S.A.** *All authors were involved in drafting the manuscript*.

## Disclosure

**J.B.L., L.B**., and **M.S.A**. are employees of RTI Health Solutions, an independent, non‐profit research institute, which received funding from Takeda for the conduct of this study. **C.A.‐S**. was an employee of Takeda Development Center Americas Inc. and a Takeda shareholder at the time of the study and is now an employee of Gilead Sciences. **Z.H., H.A., W.S., B.K**., and **J.Z**. are employees of Takeda Development Center Americas Inc. and are Takeda shareholders.

## Ethics Statement

This study involving secondary analysis of deidentified data was determined not to be research involving human subjects by the Institutional Review Board of RTI International. Individual‐level consent was not required.

## Supporting information


Data S1.

## Data Availability

The data that support the findings of this study are available from Optum and Merative, but restrictions apply to the availability of these data, which were used under license for the current study and are not publicly available.
