# Supplementary material for: A Comparative Real‐World Study Evaluating the Safety of Immune Globulin Infusion (Human) 10% Solution and Other Intravenous Immunoglobulin Therapies for the Treatment of Chronic Inflammatory Demyelinating Polyradiculoneuropathy
Source: Pharmacoepidemiol Drug Saf. 2025 Mar 17;34(3):e70124. doi: 10.1002/pds.70124 (PMC11912303; doi:10.1002/pds.70124)
Supplement: Supplementary file 1 — Data S1. [file PDS-34-e70124-s001.docx]

# A comparative real-world study evaluating the safety of immune globulin infusion (human) 10% solution and other intravenous immunoglobulin therapies for the treatment of chronic inflammatory demyelinating polyradiculoneuropathy

J. Bradley Layton,^1^ Colin Anderson-Smits,^2^* Zhongwen Huang,^2^ Hakan Ay,^2^ William Spalding,^2^ Bilal Khokhar,^2^ Jie Zhou,^2^ Lee Bennett,^1^ Mary S. Anthony^1^

*^1^RTI Health Solutions, Research Triangle Park, NC, USA; ^2^Takeda Development Center Americas, Inc., Cambridge, MA, USA.*

**at the time of the study. Current affiliation: Gilead Sciences, 333 Lakeside Dr., Foster City, CA, 94404, USA*

# Supplementary information

## SUPPLEMENTARY TEXT 1. Eligibility criteria.

*Immunoglobulin-naive cohort*

Inclusion criteria

Continuous eligible insurance plan coverage for a minimum of 183 days prior to the index date. Includes medical and pharmacy coverage. Gaps in enrollment of 31 days or less were permitted.

Diagnosis of CIDP, defined as at least two claims with recorded diagnoses of CIDP (in any coding position) separated by at least 14 days at any point on or before the index date.

Exclusion criteria

Previous use of any study IVIG therapies, non-study IVIG therapies, subcutaneous Ig therapies, or brand-unspecified Ig therapies.

Aged less than 18 years.

Missing sex information.

Use of two Ig therapies on the index date.

Diagnosis of other conditions treated with IVIG: primary immunodeficiency disease, hematologic immunodeficiency, idiopathic thrombocytopenic purpura, dermatomyositis or polymyositis, systemic sclerosis/scleroderma, myasthenia gravis or treatment with rituximab.

*Immunoglobulin-experienced cohort*

Inclusion criteria

Previous use of any study IVIG therapies, non-study IVIG therapies, subcutaneous Ig therapies, or brand-unspecified Ig therapies.

Continuous eligible insurance plan coverage for a minimum of 183 days prior to the index date. Includes medical and pharmacy coverage. Gaps in enrollment of 31 days or less were permitted.

Diagnosis of CIDP, defined as at least two claims with recorded diagnoses of CIDP (in any coding position) separated by at least 14 days at any point on or before the index date.

Exclusion criteria

Prior use of the study IVIG therapy initiated on the index date.

Aged less than 18 years.

Missing sex information.

Use of two Ig therapies on the index date. If there were multiple eligible index dates, only the first index date was retained.

Diagnosis of other conditions treated with IVIG: primary immunodeficiency disease, hematologic immunodeficiency, treatment with rituximab, idiopathic thrombocytopenic purpura, dermatomyositis or polymyositis, systemic sclerosis/scleroderma, myasthenia gravis or treatment with rituximab.

###

## SUPPLEMENTARY TEXT 2. Statistical homogeneity assessment.

The homogeneity of the HRs for each outcome from the Ig-naive and Ig-experienced cohorts was evaluated using the *I*^2^ statistic.^1–3^ If the proportionality assumption was violated, the day 365 RRs or day 365 RDs were used instead. If *I*^2^ was less than 50%, the Ig-naive and Ig-experienced cohorts were combined.

The heterogeneity across data sources of the HR, day 365 RR, and day 365 RD was evaluated using the *I*^2^ statistic. If *I*^2^ was less than 50%, the data source-specific estimates were meta-analyzed using fixed-effects meta-analytic methods.^4^

**References**

1. Higgins JP, Thompson SG. Quantifying heterogeneity in a meta-analysis. *Stat Med.* 2002;**21**(11): 1539-1558.

2. Deeks K, Higgins J, Altman D. Chapter 10: Analysing data and undertaking meta-analyses. In: Higgins. JPT, Thomas J, Chandler J, et al., eds. *Cochrane handbook for systematic reviews of interventions version 6.2.* Cochrane; 2021.

3. Huedo-Medina TB, Sanchez-Meca J, Marin-Martinez F, Botella J. Assessing heterogeneity in meta-analysis: Q statistic or I2 index? *Psychol Methods.* 2006;**11**(2): 193-206.

4. Borenstein M, Hedges LV, Higgins JP, Rothstein HR. A basic introduction to fixed-effect and random-effects models for meta-analysis. *Res Synth Methods.* 2010;**1**(2): 97-111.

## SUPPLEMENTARY TEXT 3. Quantitative bias analyses for differential outcome misclassification.

Misclassification of outcome events may result in bias of the estimated effect measure estimates, particularly if the misclassification is differential between treatment groups. Validated outcome algorithms were used wherever possible, and many of the primary outcomes have been validated in administrative claims-based populations of IVIG users with promising measures of validity.^1–3^ Differential outcome misclassification may occur if outcomes were more likely to be diagnosed accurately in one treatment group than in the other group (e.g. potentially owing to different qualities of healthcare or patterns of healthcare utilization). Feasibility evaluations and the results of this study demonstrated minimal differences in demographic characteristics and almost no differences in clinical characteristics between users of GGL and other IVIGs.^4^ Given the interchangeability of many IVIGs for CIDP treatment, differential outcome misclassification between IVIGs seems unlikely. However, because treatment group-specific positive predictive values (PPVs) are unknown, a quantitative bias analysis used a range of differential misclassification scenarios (treatment group-specific PPVs of 10–100%) to estimate the extent of potential differential outcome misclassification necessary to substantively alter the study conclusions.^5^

Quantitative bias analyses were performed in the outcome-specific analysis sets in the combined cohort. For all primary outcomes, the 1-year time period–specific RR was used, and ‘corrected’ RRs were estimated for all possible combinations of treatment group-specific PPVs as follows:^6^

$${RR}_{Corrected} = {RR}_{Observed} x\frac{{PPV}_{GGL}}{{PPV}_{Comparator}}x\frac{{Sensitivity}_{GGL}}{{Sensitivity}_{Comparator}}$$

The sensitivity of both groups was assumed to be equivalent, and thus the sensitivity term dropped out of the model. The matrix of potential corrected RR values was plotted, and the range of differential misclassification values necessary to substantively change the primary results was evaluated (as was whether those ranges of misclassification were reasonable given the published overall PPVs for the outcomes).

Plots of the potential effects of differential outcome misclassification on the 1-year RR are shown for thrombotic events (Optum Clinformatics Data Mart [Optum] combined cohort, **Supplementary** **Figure 2**; Merative MarketScan combined cohort, **Supplementary** **Figure 3**), for acute kidney injury (Optum combined cohort, **Supplementary** **Figure 4**; MarketScan combined cohort, **Supplementary Figure 5**), and for hemolytic events (MarketScan combined cohort, **Supplementary** **Figure 6**).

Across outcomes, the differences between the outcome PPV in the exposure and comparator group necessary to substantively change the study conclusion were generally much smaller at lower PPVs, with much larger differences required with higher overall PPVs. Previous validation studies of many of these outcomes have suggested that the primary study outcomes have overall PPVs (not separated by exposure status) of 60% to greater than 90%. With these known overall PPVs, at reasonable levels of assumed differential outcome misclassification (e.g. a PPV of 80% in the GGL group and 60% in the exposure group), few of the conclusions of the main analyses would be changed. However, for outcomes with RRs from the main analysis close to the null or slightly above the null (e.g. thrombotic events in MarketScan [RR = 1.14], AKI in MarketScan [RR = 1.25]), differential misclassification of 20% could potentially result in a true RR of greater than 1.5.

**References**

1. Ammann EM, Leira EC, Winiecki SK, et al. Chart validation of inpatient ICD-9-CM administrative diagnosis codes for ischemic stroke among IGIV users in the Sentinel Distributed Database. *Medicine (Baltimore).* 2017;**96**(52): e9440.

2. Ammann EM, Schweizer ML, Robinson JG, et al. Chart validation of inpatient ICD-9-CM administrative diagnosis codes for acute myocardial infarction (AMI) among intravenous immune globulin (IGIV) users in the Sentinel Distributed Database. *Pharmacoepidemiol Drug Saf.* 2018;**27**(4): 398-404.

3. Ammann EM, Cuker A, Carnahan RM, et al. Chart validation of inpatient International Classification of Diseases, Ninth Revision, Clinical Modification (ICD-9-CM) administrative diagnosis codes for venous thromboembolism (VTE) among intravenous immune globulin (IGIV) users in the Sentinel Distributed Database. *Medicine (Baltimore).* 2018;**97**(8): e9960.

4. Anderson-Smits C, Layton JB, Ritchey ME et al. Patient and treatment characteristics of a large US sample of patients with chronic inflammatory demyelinating polyradiculoneuropathy (CIDP) initiating intravenous immunoglobulin (IVIG) therapy (4212). *Neurology* 2021;**96**(15 Supplement): 4212.

5. Brenner H, Gefeller O. Use of the positive predictive value to correct for disease misclassification in epidemiologic studies. *Am J Epidemiol.* 1993;**138**(11): 1007-1015.

6. Newcomer SR, Xu S, Kulldorff M, Daley MF, Fireman B, Glanz JM. A primer on quantitative bias analysis with positive predictive values in research using electronic health data. *J Am Med Inform Assoc.* 2019;**26**(12): 1664-1674.

## SUPPLEMENTARY TEXT 4. Subgroup analyses.

In Optum Clinformatics Data Mart, no acute VTE cases occurred among women in the comparator group. However, because VTE cases accounted for most of the thrombotic events, this difference contributed to the numerically increased HR observed for thrombotic events for women (HR, 6.39 [95% CI, 0.98–41.60]; Figure 5), although this HR was very imprecise owing to the small number of cases with unequal distribution between treatment groups (5 GGL cases and 1 comparator case). This pattern was not observed in MarketScan.

## SUPPLEMENTARY TABLE 1. Safety outcomes considered in this study, outcome-specific exclusion criteria and previous outcome validation.

| **Safety outcome** | **Outcome algorithm** | **Outcome-specific exclusion criteria** | **Previous validation** |
| --- | --- | --- | --- |
| Thrombotic events | Diagnosis of AIS, AMI, or VTE on an inpatient facility claim, as defined for each of the following individual thrombotic event subtypes:  AIS diagnosis in the principal coding position on an inpatient facility claim  AMI diagnosis in any coding position on an inpatient facility claim  VTE (including DVT, PE, or CVT) diagnosis in any coding position on an inpatient facility claim | AIS, AMI, or VTE diagnosis in any coding position or setting at any time before the index date | Thrombotic events were validated by FDA investigators in IVIG users (although not necessarily with CIDP) in the Sentinel Initiative^1–3^  PPV for AIS = 64% (95% CI 35–87%) for inpatient facility claims for AIS in the principal coding position after excluding those with previous AIS  PPV for AMI = 92% (95% CI 74–99%) for inpatient claims in the principal coding position; 96% (95% CI, 78–100%) for secondary coding positions after excluding those with previous AMI  PPV for VTE = 93% (95% CI 76–99%) for inpatient claims in the principal coding position; 100% (95% CI 29–100%) for secondary coding positions after excluding those with previous VTE |
| AKI | AKI diagnosis in any coding position or setting | AKI diagnosis in any coding position or setting at any time before the index date  End-stage renal disease diagnosis in any coding position or setting at any time before the index date  Procedure code for dialysis at any time before the index date | Validated by FDA investigators in the Sentinel Initiative^4^  PPV for AKI = 85% (95% CI 78–91%) |
| Hemolytic events | Hemolysis diagnosis in any coding position or setting | Hemolytic event in any coding position or setting at any time before index date  Hereditary hemolytic anemia diagnosis of known etiology in any coding position or setting at any time before index date | Not formally validated but follows a recommended algorithm developed by CBER^5^ |

Abbreviations: AIS, acute ischemic stroke; AKI, acute kidney injury; AMI, acute myocardial infarction; CBER, Center for Biologics Evaluation and Research; CI, confidence interval; CVT, cerebral venous thrombosis; DVT, deep vein thrombosis; FDA, US Food and Drug Administration; PE, pulmonary embolism; PPV, positive predictive value; VTE, acute venous thromboembolism.

**References**

1. Ammann EM, Leira EC, Winiecki SK, et al. Chart validation of inpatient ICD-9-CM administrative diagnosis codes for ischemic stroke among IGIV users in the Sentinel Distributed Database. *Medicine (Baltimore).* 2017;**96**(52): e9440.

2. Ammann EM, Schweizer ML, Robinson JG, et al. Chart validation of inpatient ICD-9-CM administrative diagnosis codes for acute myocardial infarction (AMI) among intravenous immune globulin (IGIV) users in the Sentinel Distributed Database. *Pharmacoepidemiol Drug Saf.* 2018;**27**(4): 398-404.

3. Ammann EM, Cuker A, Carnahan RM, et al. Chart validation of inpatient International Classification of Diseases, Ninth Revision, Clinical Modification (ICD-9-CM) administrative diagnosis codes for venous thromboembolism (VTE) among intravenous immune globulin (IGIV) users in the Sentinel Distributed Database. *Medicine (Baltimore).* 2018;**97**(8): e9960.

4. Patel U, Smith D, Gurwitz J, Hsu C, Parikh C, Brunelli S, et al. Mini-Sentinel Coordinating Center. Validation of acute kidney injury cases in the Mini-Sentinel Distributed Database. 2013. Available from [https://www.sentinelinitiative.org/sites/default/files/Drugs/Assessments/Mini-Sentinel_Validation-of-Acute-Kidney-Injury-Cases.pdf. Accessed 6 May 2022](https://www.sentinelinitiative.org/sites/default/files/Drugs/Assessments/Mini-Sentinel_Validation-of-Acute-Kidney-Injury-Cases.pdf.%20Accessed%206%20May%202022).

5. Saunders-Hastings P, Burrell T, Srichaikul J, Dores G, Chada K, Wong HL, et al. US Food and Drug Administration, Center for Biologics Evaluation and Research, Office of Biostatistics and Epidemiology. Defining acquired hemolysis using administrative claims data: a case algorithm 2020. Available from [https://www.bestinitiative.org/wp-content/uploads/2020/08/Hemolysis_Algorithm_Report_2020.pdf. Accessed 4 August 2021](https://www.bestinitiative.org/wp-content/uploads/2020/08/Hemolysis_Algorithm_Report_2020.pdf.%20Accessed%204%20August%202021).

## SUPPLEMENTARY TABLE 2. Covariates included in outcome-specific propensity score models.

| Variable | Assessment period (days relative to index date) | Outcome | | |
| --- | --- | --- | --- | --- |
|  |  | Thrombotic events | AKI | Hemolytic events |
| **Demographic characteristics** |  |  |  |  |
| Age (linear, squared, and cubic terms) | [0] | X | X | X |
| Sex (categorical: female, male) | [0] | X | X | X |
| Calendar year of IVIG initiation (categorical) | [0] | X | X | X |
| Geographic region (categorical) | [0] | X | X | X |
| Insurance type (categorical: commercial or noncommercial [Medicaid/Medicare]) | [0] | X | X | X |
| Race/ethnicity (categorical: Black or African American, White, or another race/ethnicity or unknown [combining all other stated races, unspecified race, and missing race]) (only in Optum) | [0] | X | X | X |
| Education level (categorical) (only in Optum) | [0] | X | X | X |
| **Healthcare utilization** |  |  |  |  |
| Cancer screening | [−183, −1] | X | X | X |
| Clinic visits (0–2, 3–5, ≥ 6) (categorical) | [−183, −1] | X | X | X |
| Hospitalizations (0, 1, ≥ 2) (categorical) | [−183, −1] | X | X | X |
| ED visits (0, 1, ≥ 2) (categorical) | [−183, −1] | X | X | X |
| Systemic anti-infective agent use | [−183, −1] | X | X | X |
| **Comorbidities** |  |  |  |  |
| Hypertension | [−all available, −1] | X | X | X |
| Heart failure | [−all available, −1] | X | X | X |
| Cardiac arrhythmia | [−all available, −1] | X | X | X |
| AMI, history | [−all available, −1] |  | X | X |
| Other cardiovascular disease | [−all available, −1] | X | X | X |
| Ischemic stroke or transient ischemic attack | [−all available, −1] |  | X | X |
| Venous thromboembolism, history | [−all available, −1] |  | X | X |
| Peripheral vascular disease | [−all available, −1] | X | X | X |
| Hemolysis | [−all available, −1] | X | X |  |
| Cancer | [−all available, −1] | X | X | X |
| Diabetes mellitus (type 1 or 2) | [−all available, −1] | X | X | X |
| Autoimmune disorders (psoriasis, inflammatory bowel disease, Graves’ disease, Sjögren’s syndrome, Hashimoto’s thyroiditis, autoimmune vasculitis, celiac disease, unspecified autoimmune disease, rheumatic heart disease, sarcoidosis, systemic lupus erythematosus) | [−all available, −1] | X | X | X |
| Pre-existing renal disease (chronic kidney disease or renal disorders, end-stage renal disease, receipt of dialysis) | [−all available, −1] | X | X | X |
| AKI^a^ | [−all available, −1] | X |  | X |
| Liver disease or jaundice | [−all available, −1] | X | X | X |
| Pancreatitis | [−all available, −1] | X | X |  |
| Anemia (hereditary hemolytic anemia or other anemia) | [−all available, −1] | X | X | X |
| Adrenal insufficiency | [−all available, −1] | X | X | X |
| Acquired hypothyroidism | [−all available, −1] | X | X | X |
| Serious infection | [−all available, −1] | X | X | X |
| Chronic pulmonary disease | [−all available, −1] | X | X | X |
| Peptic ulcer disease | [−all available, −1] |  | X |  |
| Hemiplegia/paraplegia | [−all available, −1] | X | X |  |
| HIV/AIDS | [−all available, −1] |  |  | X |
| Anaphylaxis^a^ | [−all available, −1] |  |  | X |
| TRALI | [−all available, −1] |  | X | X |
| TACO/fluid overload | [−all available, −1] |  | X | X |
| **Comedications** |  |  |  |  |
| Cardiovascular medications (anticoagulants, antihypertensives, lipid-lowering drugs) | [−all available, −1] | X | X | X |
| Opioids | [−all available, −1] | X | X | X |
| Oral contraceptives | [−all available, −1] | X | X | X |
| High-dose systemic corticosteroid use | [−all available, −1] | X | X | X |
| Plasma exchange and/or plasmapheresis | [−all available, −1] | X | X | X |
| Immunomodulatory agent use | [−all available, −1] | X | X | X |
| Related conditions or diagnoses |  |  |  |  |
| Other neuropathies (hereditary and idiopathic neuropathy, MMN and other inflammatory neuropathies, drug-induced polyneuropathy, diabetic neuropathy) | [−all available, −1] | X | X | X |
| Parkinson’s disease | [−all available, −1] | X | X | X |
| Multiple sclerosis/transverse myelitis | [−all available, −1] | X | X | X |
| **Indicators of severity or functional status** |  |  |  |  |
| Neuropathic or chronic pain | [−all available, −1] | X | X | X |
| Difficulty walking | [−all available, −1] | X | X | X |
| Abnormal nerve function | [−all available, −1] | X | X | X |
| Weakness | [−all available, −1] | X | X | X |
| Use of a wheelchair or walking aid | [−all available, −1] | X | X | X |
| Falls | [−all available, −1] | X | X | X |
| Fractures | [−all available, −1] | X | X | X |
| Stay in a skilled nursing facility | [−all available, −1] | X | X | X |
| **CIDP diagnostic workup** |  |  |  |  |
| Electrodiagnostic nerve study | [−all available, −1] | X | X | X |
| Nerve biopsy | [−all available, −1] | X | X | X |
| Magnetic resonance imagining | [−all available, −1] | X | X | X |
| Spinal fluid testing | [−all available, −1] | X | X | X |
| Serum Ig testing | [−all available, −1] | X | X | X |
| **Markers of dependence in activities of daily living** |  |  |  |  |
| Home medical equipment (home hospital bed, home oxygen) | [−all available, −1] | X | X | X |
| Ambulance/life support | [−all available, −1] | X | X | X |
| Lipid abnormality | [−all available, −1] | X | X | X |
| Psychiatric disorder | [−all available, −1] | X | X | X |
| Vertigo | [−all available, −1] | X | X | X |
| Arthritis | [−all available, −1] | X | X | X |
| Burns | [−all available, −1] | X | X | X |
| Ig-experienced and combined cohorts, only |  |  |  |  |
| Number of unique Ig therapies used before the index date (0, 1, ≥ 2) (categorical) | [−all available, −1] | X | X | X |
| Timing of use of other Ig therapies before the index date (categorical) | [−all available, −91]  [−90, −31]  [−30, −1] | X | X | X |

Abbreviations: AIDS, acquired immunodeficiency syndrome; AKI, acute kidney injury; AMI, acute myocardial infarction; CIDP, chronic inflammatory demyelinating polyneuropathy; ED, emergency department; HIV, human immunodeficiency virus; Ig, immunoglobulin; IVIG, intravenous immunoglobulin; MMN, multifocal motor neuropathy; TACO, transfusion-associated circulatory overload; TRALI, transfusion-related acute lung injury.

X indicates that the variable was considered for inclusion in the analysis-specific propensity score model. Owing to the sparseness of some covariates potentially resulting in convergence issues of propensity score models, candidate variables that were not present in at least 1% of participants in each exposure group (or were present in > 99% of participants in each exposure group) were not included in an outcome-specific propensity score model. Additionally, variables that were not present in at least 2% of participants in each exposure group (or were present in > 98% of participants in each exposure group) and had absolute standardized difference values of 0.1 or less in the crude cohort were not included in the outcome-specific propensity score model because these variables were rare (or common) and were well balanced between groups, and thus were unlikely to be informative in the propensity score models.

^a^Those with recent histories of these outcomes (anaphylaxis, burns) before the index date were excluded from the outcome-specific analytic cohorts after application of the outcome-specific washout periods; however, those with “any history” of these outcomes were retained because they indicate more distant histories of anaphylaxis or burns than those used to define the washout criterion.

## SUPPLEMENTARY TABLE 3. Crude and propensity score–weighted hazard ratios for GGL versus comparator IVIGs for components of the thrombotic events composite outcome for the combined cohort.

| Thrombotic event | Treatment group | N | Cases | Crude IR (95% CI)^a^ | Crude HR (95% CI) | Weighted HR (95% CI) |
| --- | --- | --- | --- | --- | --- | --- |
| Optum |  |  |  |  |  |  |
| Composite | GGL | 489 | 15 | 47.8  (26.7, 78.8) | 1.68  (0.85, 3.34) | 1.52  (0.75, 3.07) |
|  | Comparator | 969 | 18 | 28.9  (17.1, 45.6) | — | — |
| AIS | GGL | 489 | 0 | 0  (0, 11.4) | NE | NE |
|  | Comparator | 969 | 3 | 4.8  (1.0, 14.0) | — | — |
| AMI | GGL | 489 | 3 | 9.3  (1.9, 27.2) | 2.06  (0.41, 10.19) | 1.73  (0.34, 8.80) |
|  | Comparator | 969 | 3 | 4.8  (1.0, 14.0) | — | — |
| VTE | GGL | 489 | 12 | 38.1  (19.7, 66.6) | 1.85  (0.84, 4.05) | 1.64  (0.73, 3.72) |
|  | Comparator | 969 | 13 | 20.8  (11.1, 35.5) | — | — |
| MarketScan |  |  |  |  |  |  |
| Composite | GGL | 1165 | 17 | 23.0  (13.4, 36.8) | 1.07  (0.59, 1.95) | 1.23  (0.66, 2.29) |
|  | Comparator | 2225 | 30 | 21.7  (14.7, 31.0) | — | — |
| AIS | GGL | 1165 | 2 | 2.7  (0.3, 9.6) | NE | NE |
|  | Comparator | 2225 | 1 | 0.7  (0.0, 4.0) | — | — |
| AMI | GGL | 1165 | 6 | 8.0  (2.9, 17.4) | 1.98  (0.64, 6.13) | 2.29  (0.70, 7.50) |
|  | Comparator | 2225 | 6 | 4.3  (1.6, 9.4) | — | — |
| VTE | GGL | 1165 | 9 | 12.1  (5.5, 23.0) | 0.73  (0.34, 1.59) | 0.82  (0.36, 1.84) |
|  | Comparator | 2225 | 23 | 16.6  (10.5, 25.0) | — | — |

— indicates the reference group.

Note: The combined cohort was not analyzed for AIS in either database.

^a^IR estimates scaled as events per 1000 person-years.

Abbreviations: AIS, acute ischemic stroke; AMI, acute myocardial infarction; CI, confidence interval; GGL, GAMMAGARD LIQUID; HR, hazard ratio; IR, incidence rate; IVIG, intravenous immunoglobulin; NE, not estimated; VTE, venous thromboembolism.

## SUPPLEMENTARY FIGURE 1. Cohort eligibility, covariate assessment, and follow-up windows relative to IVIG initiation for the Ig-naive (A) and Ig-experienced cohorts (B)


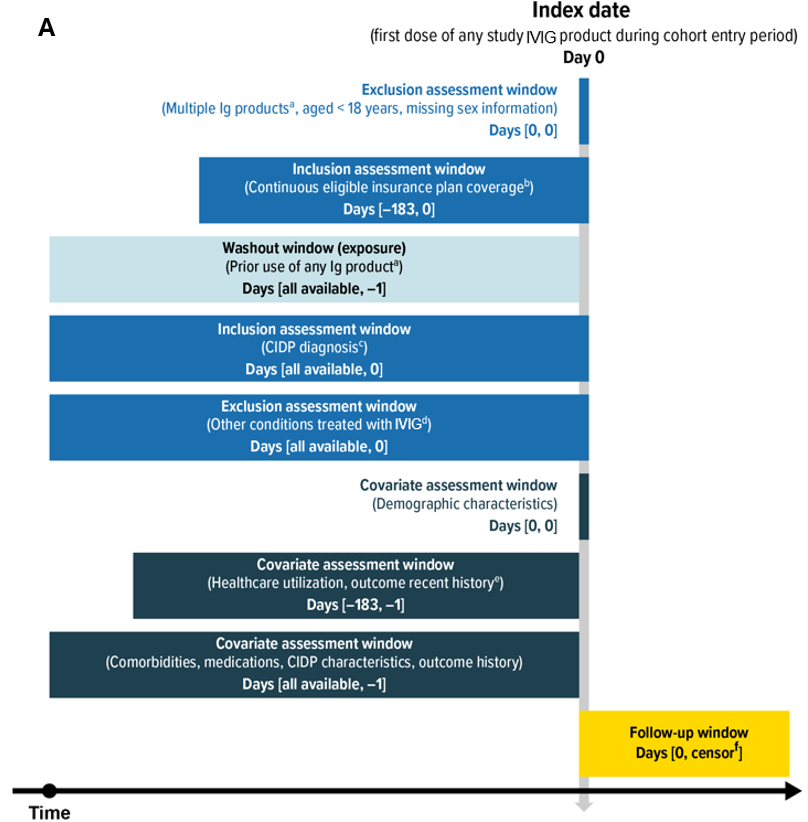


^a^Any study IVIGs, non-study IVIGs, subcutaneous Ig, or brand-unspecified Ig therapies.

^b^Continuous eligible enrollment includes medical and pharmacy coverage. Gaps in enrollment of ≤ 31 days are permitted.

^c^At least 2 claims with recorded diagnoses of CIDP (in any coding position) separated by at least 14 days.

^d^Primary immunodeficiency disease, hematologic immunodeficiency, treatment with rituximab, idiopathic thrombocytopenic purpura, dermatomyositis or polymyositis, systemic sclerosis/scleroderma, myasthenia gravis.

^e^Anaphylaxis and burns, which have washout periods of 183 days.

^f^Study outcome, 31 December 2019 (end of the study period), disenrollment from eligible coverage, end of continuous use of index IVIG product, or switching to or adding a different Ig therapy (study IVIGs, non-study IVIGs, subcutaneous Ig, or brand-unspecified Ig therapies).

Abbreviations: CIDP, chronic inflammatory demyelinating polyradiculoneuropathy; Ig, immunoglobulin; IVIG, intravenous immunoglobulin.


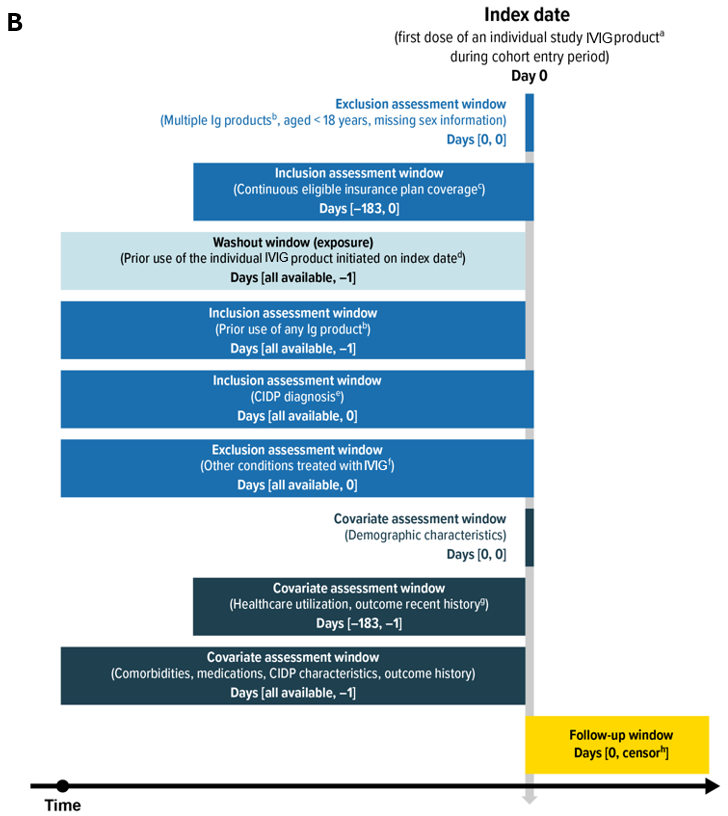


^a^The first use of each study IVIG within an individual was identified and evaluated separately; only the first IVIG initiation meeting all eligibility criteria per person was included.

^b^Any study IVIGs, non-study IVIGs, subcutaneous Ig, or brand-unspecified Ig therapies other than the IVIG initiated on the index date.

^c^Continuous eligible enrollment includes medical and pharmacy coverage. Gaps in enrollment of ≤ 31 days are permitted.

^d^To ensure initiation of the individual IVIG, only previous use of the study IVIG initiated on the index date was considered an exclusion criterion. Use of other Ig therapies did not constitute an exclusion criterion in this cohort.

^e^At least 2 claims with recorded diagnoses of CIDP (in any coding position) separated by at least 14 days.

^f^Primary immunodeficiency disease, hematologic immunodeficiency, treatment with rituximab, idiopathic thrombocytopenic purpura, dermatomyositis or polymyositis, systemic sclerosis/scleroderma; myasthenia gravis.

Abbreviations: CIDP, chronic inflammatory demyelinating polyradiculoneuropathy; Ig, immunoglobulin; IVIG, intravenous immunoglobulin.

## SUPPLEMENTARY FIGURE 2. Corrected 1-year risk ratios for thrombotic events under an array of differential outcome misclassification scenarios for the combined cohort, Optum.


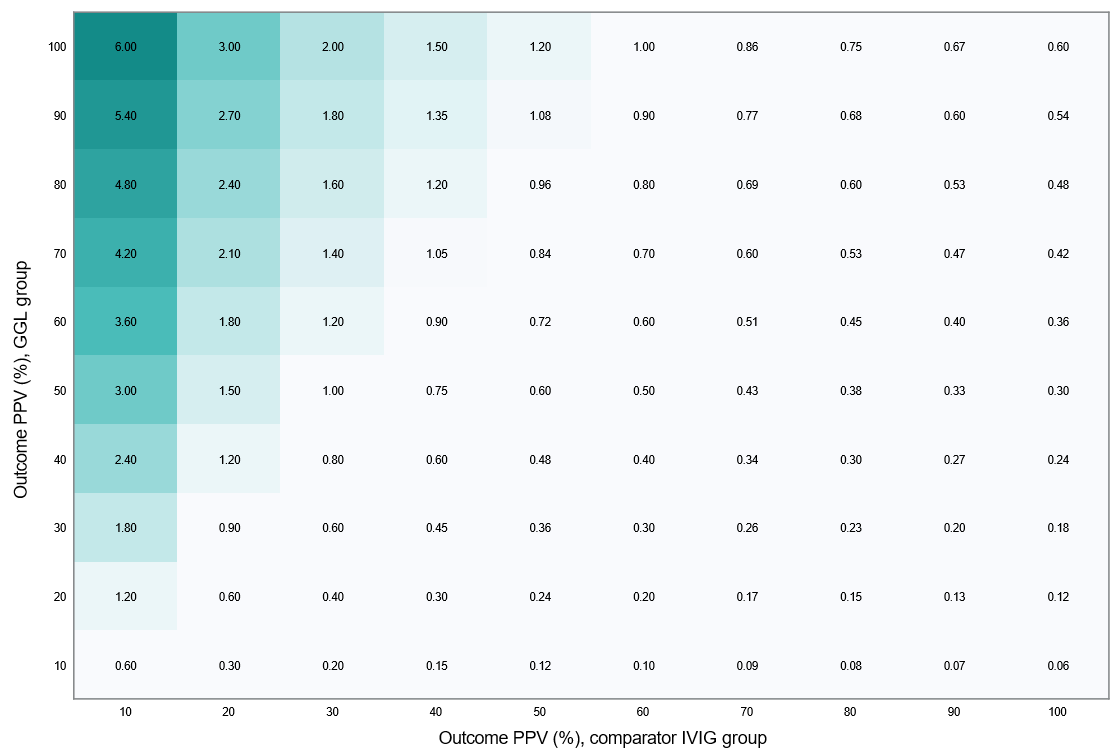


Potential outcome PPVs in the GGL group are given on the Y axis and potential outcome PPVs in the exposure group are given on the X axis. Each point on the matrix is a corrected 1-year RR estimate accounting for the particular combination of differential outcome misclassification at that point. Along the diagonal line representing nondifferential misclassification (from the intersection of 10% and 10% to the intersection of 100% and 100%), the RR estimates are all the same as the observed 1-year RR from the main analysis. In all cases, if the PPV of the outcome algorithm was higher in the GGL exposure group than in the comparator group, the corrected RR estimate would be higher than the observed estimate (i.e. it assumes that a larger proportion of the cases in the exposure group than in the comparator group were true cases). In the example thrombotic events figure, if the thrombotic events outcome PPV was 90% in the GGL group and 70% in the comparator group, the corrected RR would be 0.77, instead of the RR of 0.60 observed in the main analysis.

Abbreviations: GGL, immune globulin infusion (human) 10% solution (GAMMAGARD LIQUID); IVIG, intravenous immunoglobulin; PPV, positive predictive value; RR, relative risk.

## SUPPLEMENTARY FIGURE 3. Corrected 1-year risk ratios for thrombotic events under an array of differential outcome misclassification scenarios for the combined cohort, MarketScan.

**
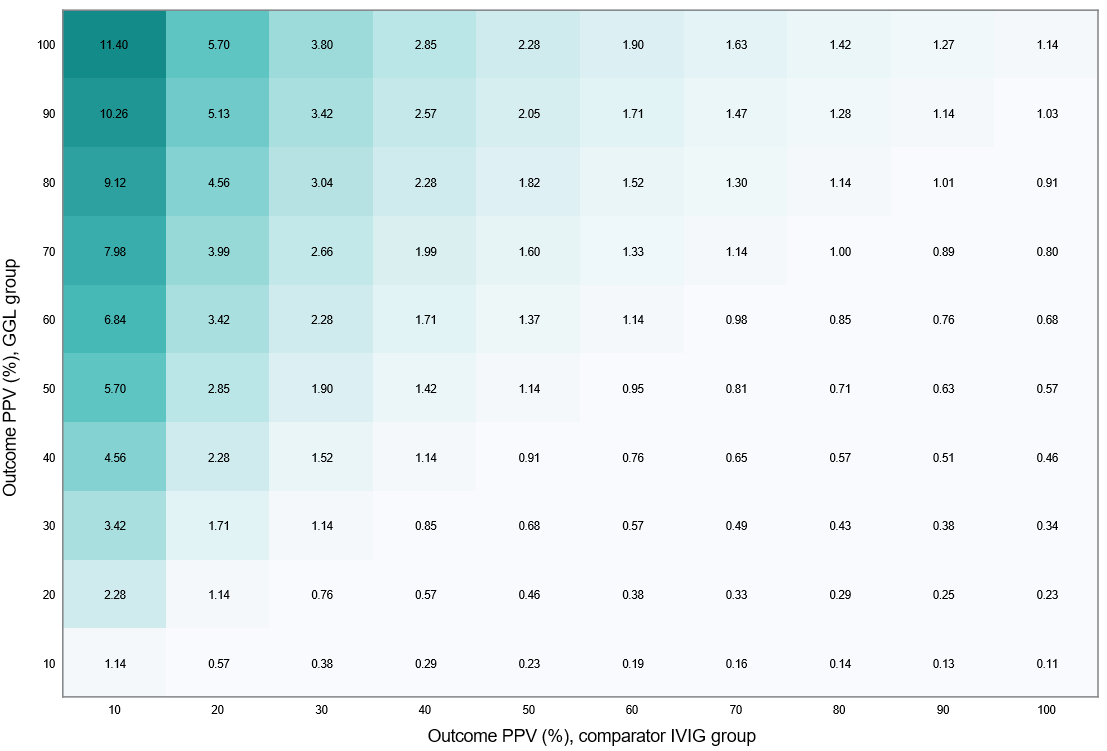
**

Potential outcome PPVs in the GGL group are given on the Y axis and potential outcome PPVs in the exposure group are given on the X axis. Each point on the matrix is a corrected 1-year RR estimate accounting for the particular combination of differential outcome misclassification at that point. Along the diagonal line representing nondifferential misclassification (from the intersection of 10% and 10% to the intersection of 100% and 100%), the RR estimates are all the same as the observed 1-year RR from the main analysis. In all cases, if the PPV of the outcome algorithm was higher in the GGL exposure group than in the comparator group, the corrected RR estimate would be higher than the observed estimate (i.e. it assumes that a larger proportion of the cases in the exposure group than in the comparator group were true cases). In the example thrombotic events figure, if the thrombotic events outcome PPV was 90% in the GGL group and 70% in the comparator group, the corrected RR would be 0.77, instead of the RR of 0.60 observed in the main analysis.

Abbreviations: GGL, immune globulin infusion (human) 10% solution (GAMMAGARD LIQUID); IVIG, intravenous immunoglobulin; PPV, positive predictive value; RR, relative risk.

## SUPPLEMENTARY FIGURE 4. Corrected 1-year risk ratios for acute kidney injury under an array of differential outcome misclassification scenarios for the combined cohort, Optum.

**
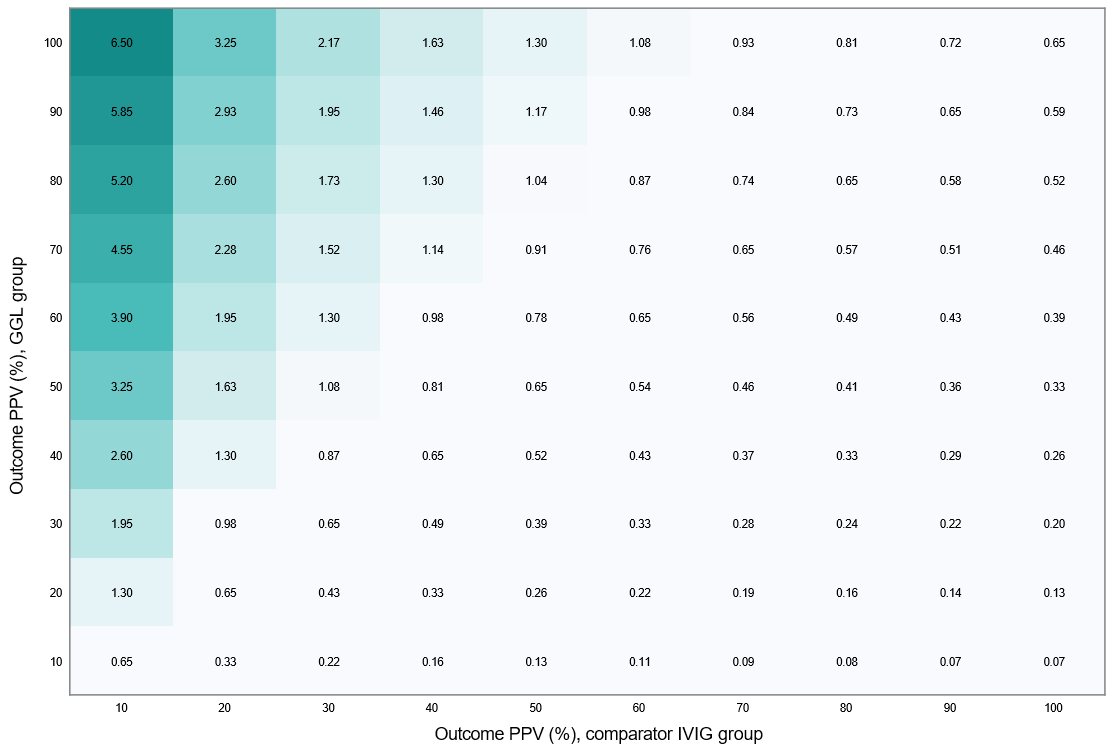
**

Potential outcome PPVs in the GGL group are given on the Y axis and potential outcome PPVs in the exposure group are given on the X axis. Each point on the matrix is a corrected 1-year RR estimate accounting for the particular combination of differential outcome misclassification at that point. Along the diagonal line representing nondifferential misclassification (from the intersection of 10% and 10% to the intersection of 100% and 100%), the RR estimates are all the same as the observed 1-year RR from the main analysis. In all cases, if the PPV of the outcome algorithm was higher in the GGL exposure group than in the comparator group, the corrected RR estimate would be higher than the observed estimate (i.e. it assumes that a larger proportion of the cases in the exposure group than in the comparator group were true cases).

Abbreviations: GGL, immune globulin infusion (human) 10% solution (GAMMAGARD LIQUID); IVIG, intravenous immunoglobulin; PPV, positive predictive value; RR, relative risk.

## SUPPLEMENTARY FIGURE 5. Corrected 1-year risk ratios for acute kidney injury under an array of differential outcome misclassification scenarios for the combined cohort, MarketScan.

**
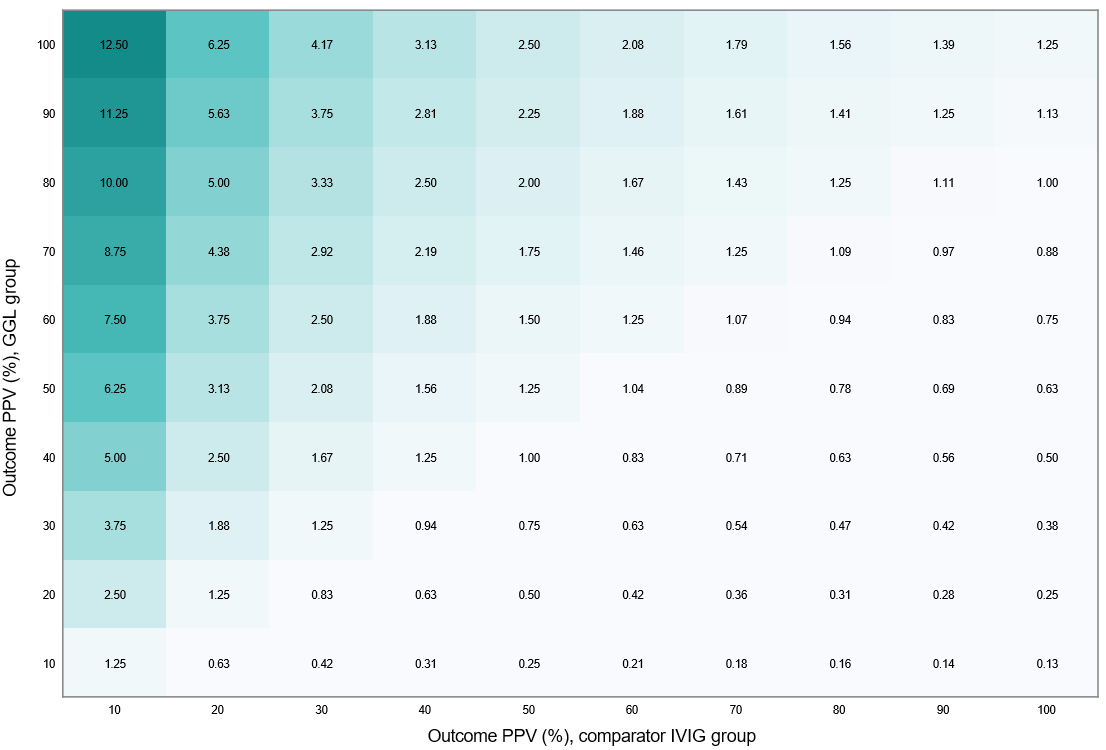
**

Potential outcome PPVs in the GGL group are given on the Y axis and potential outcome PPVs in the exposure group are given on the X axis. Each point on the matrix is a corrected 1-year RR estimate accounting for the particular combination of differential outcome misclassification at that point. Along the diagonal line representing nondifferential misclassification (from the intersection of 10% and 10% to the intersection of 100% and 100%), the RR estimates are all the same as the observed 1-year RR from the main analysis. In all cases, if the PPV of the outcome algorithm was higher in the GGL exposure group than in the comparator group, the corrected RR estimate would be higher than the observed estimate (i.e. it assumes that a larger proportion of the cases in the exposure group than in the comparator group were true cases).

Abbreviations: GGL, immune globulin infusion (human) 10% solution (GAMMAGARD LIQUID); IVIG, intravenous immunoglobulin; PPV, positive predictive value; RR, relative risk.

## SUPPLEMENTARY FIGURE 6. Corrected 1-year risk ratios for hemolytic events under an array of differential outcome misclassification scenarios, combined cohort, MarketScan.


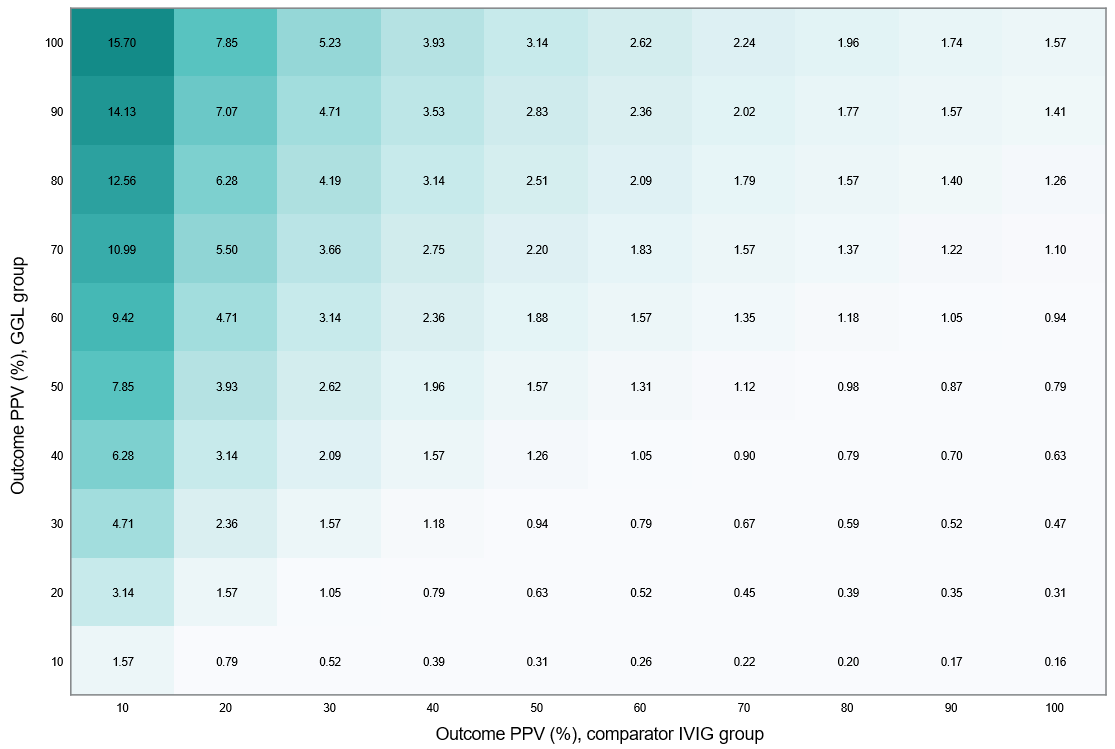


Potential outcome PPVs in the GGL group are given on the Y axis and potential outcome PPVs in the exposure group are given on the X axis. Each point on the matrix is a corrected 1-year RR estimate accounting for the particular combination of differential outcome misclassification at that point. Along the diagonal line representing nondifferential misclassification (from the intersection of 10% and 10% to the intersection of 100% and 100%), the RR estimates are all the same as the observed 1-year RR from the main analysis. In all cases, if the PPV of the outcome algorithm was higher in the GGL exposure group than in the comparator group, the corrected RR estimate would be higher than the observed estimate (i.e. it assumes that a larger proportion of the cases in the exposure group than in the comparator group were true cases).

Abbreviations: GGL, immune globulin infusion (human) 10% solution (GAMMAGARD LIQUID); IVIG, intravenous immunoglobulin; PPV, positive predictive value; RR, relative risk.

## SUPPLEMENTARY FIGURE 7. Example of unweighted and weighted absolute standardized differences in covariates: thrombotic events composite outcome analysis set, combined cohort, Optum database.


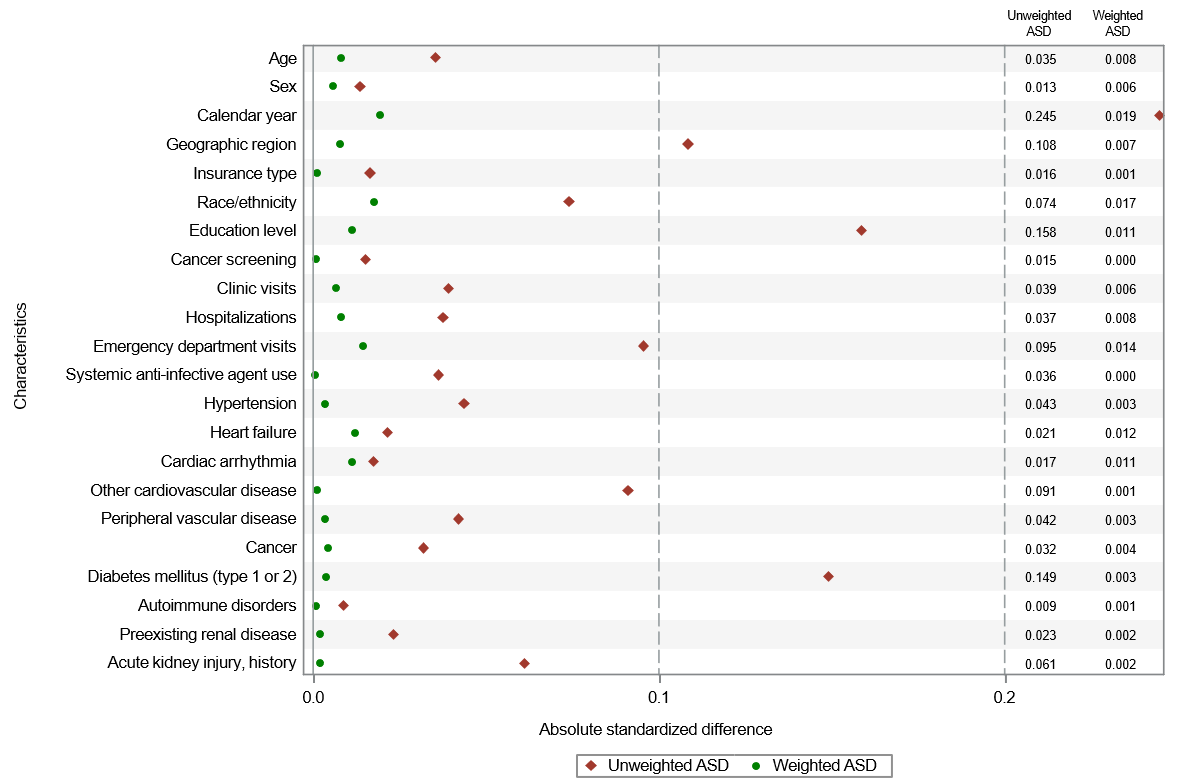


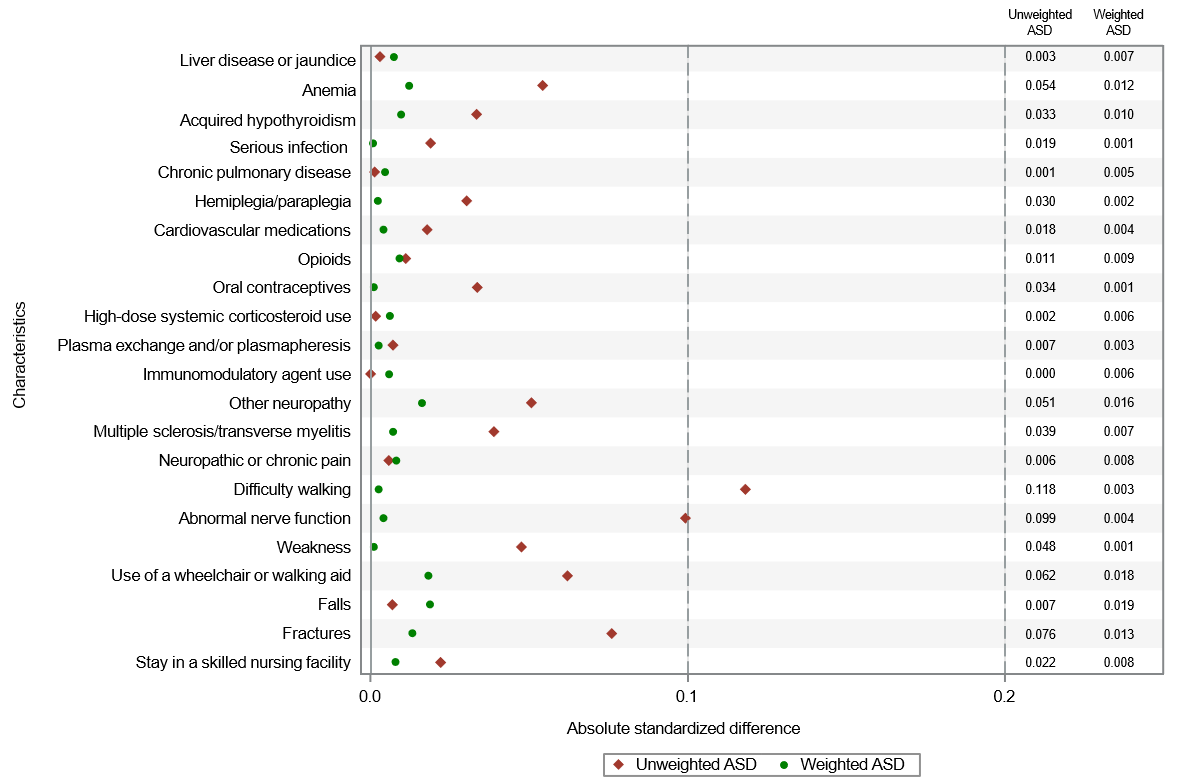


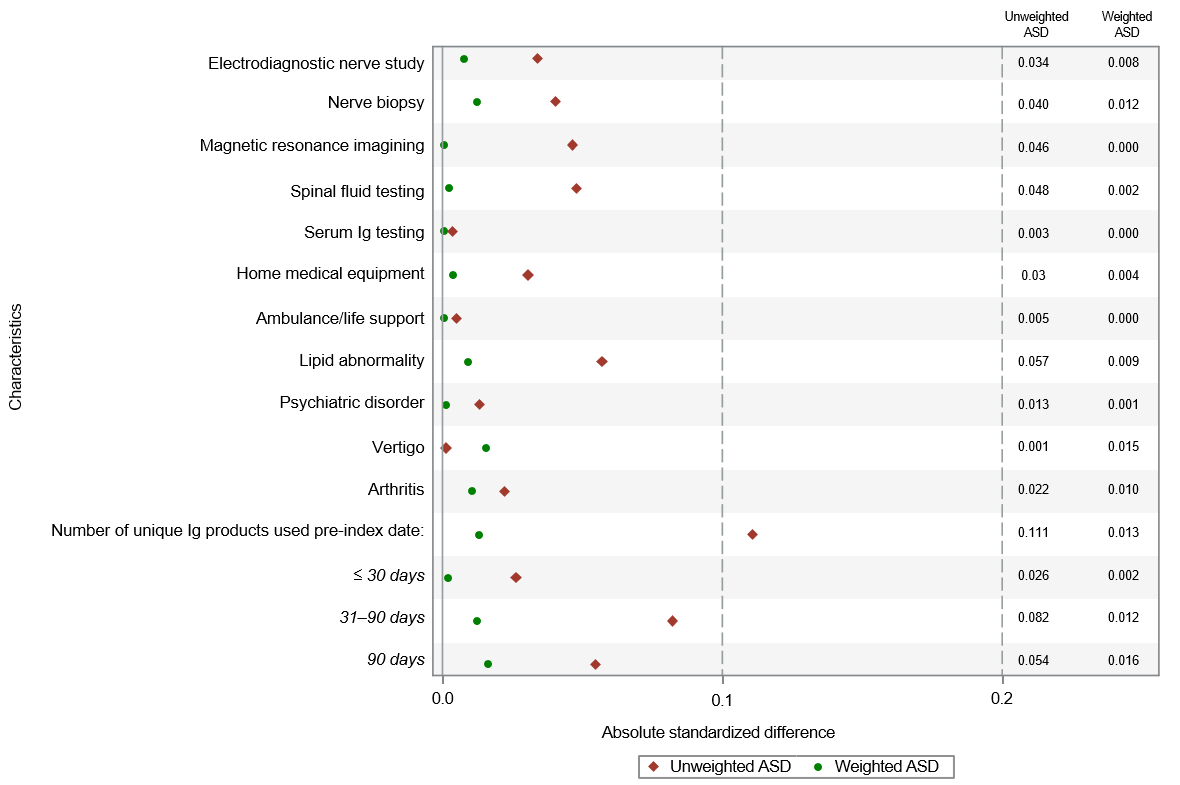


Abbreviations: ASD, absolute standardized difference, Ig, immunoglobulin.

## SUPPLEMENTARY FIGURE 8. Weighted cumulative incidence of acute kidney injury in IVIG initiators with CIDP by treatment group and data source, combined cohort.

**
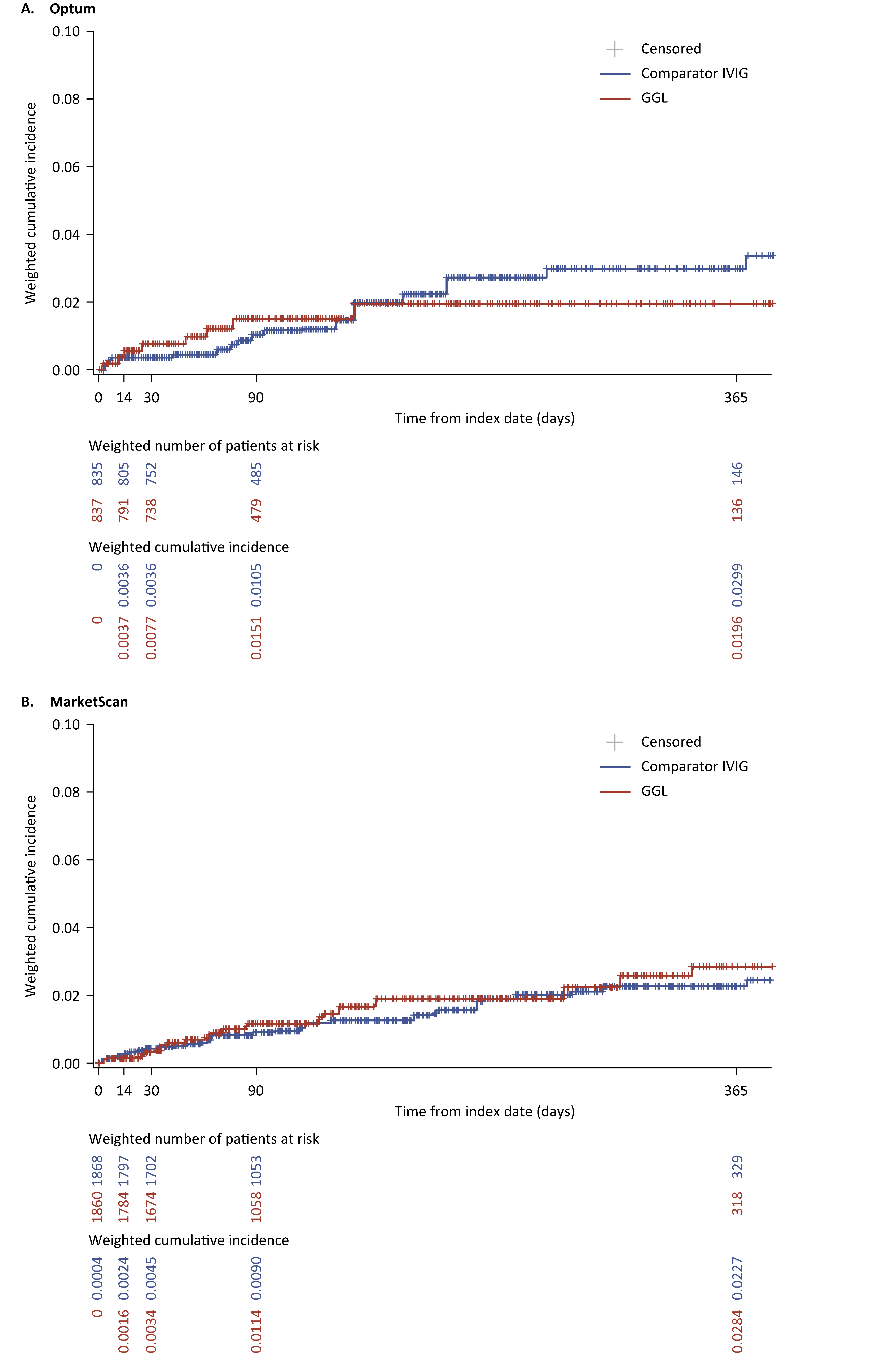
**

Abbreviations: CIDP, chronic inflammatory demyelinating polyradiculoneuropathy; GGL, immune globulin infusion (human) 10% solution (GAMMAGARD LIQUID); IVIG, intravenous immunoglobulin.

## SUPPLEMENTARY FIGURE 9. Weighted cumulative incidence of hemolytic events in IVIG initiators with CIDP by treatment group and data source.

**
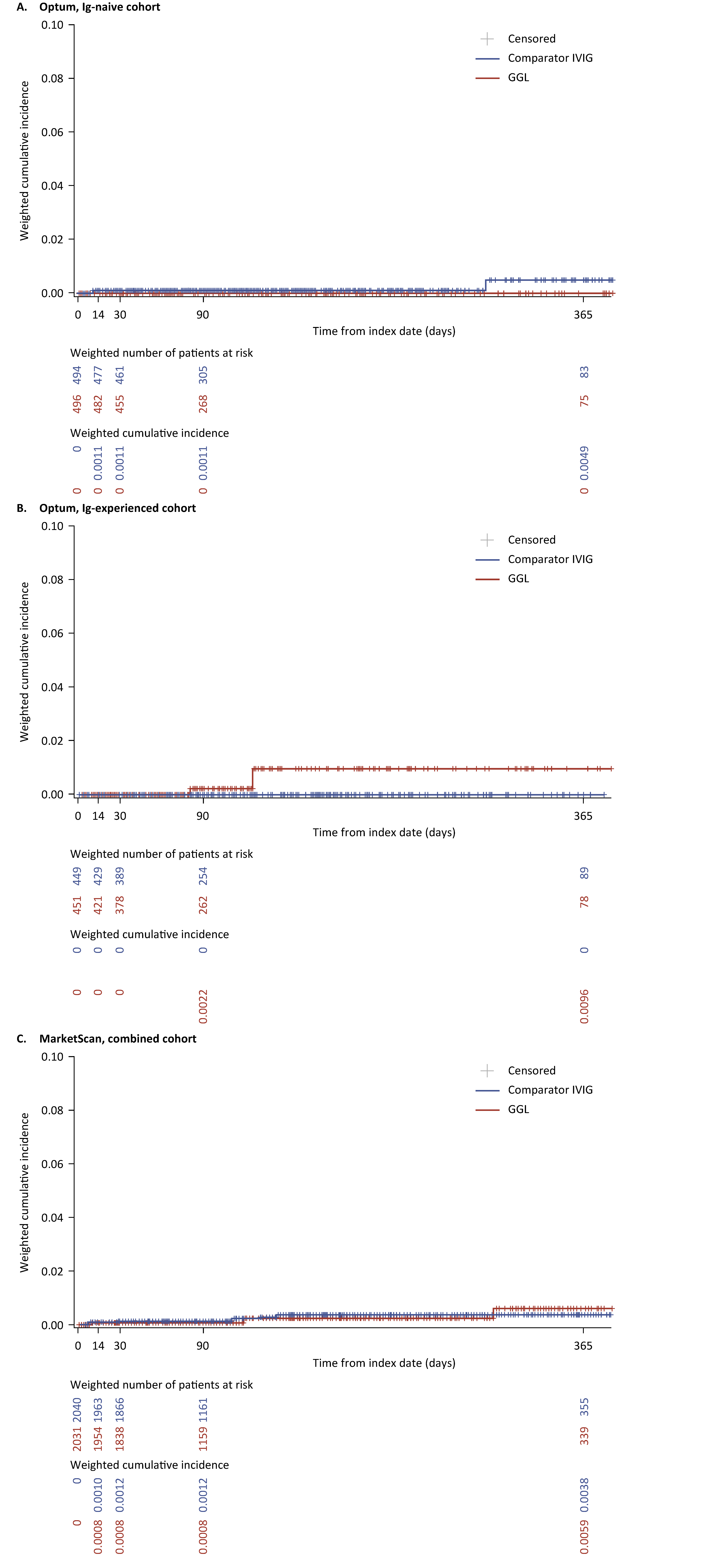
**

**
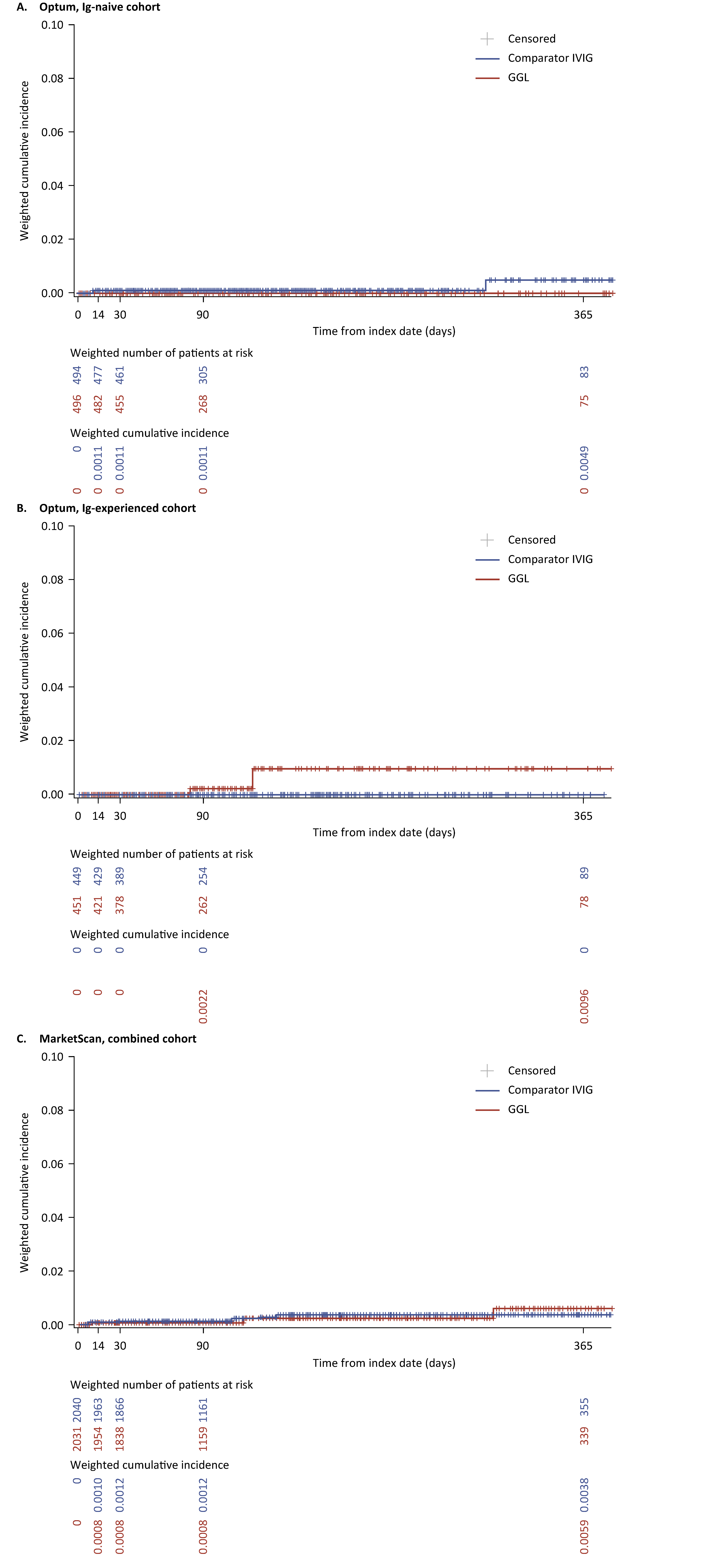
**

Abbreviations: CIDP, chronic inflammatory demyelinating polyradiculoneuropathy; GGL, immune globulin infusion (human) 10% solution (GAMMAGARD LIQUID); Ig, immunoglobulin; IVIG, intravenous immunoglobulin.
